# Exploring the Protective Effect of *Gastrodia elata* Extract on D-Galactose-Induced Liver Injury in Mice Based on the PI3K/Akt Signaling Pathway

**DOI:** 10.3390/cimb48010006

**Published:** 2025-12-20

**Authors:** Liu Han, Hongyu Zhai, Xiangyu Ma, He Li, Qiaosen Ren, Jiating Liu, Zhe Zhang, Xintong Li, Qiuyue Zhang, Xin Sun

**Affiliations:** 1 College of Pharmacy, Jilin Medical University, Jilin 132013, China; 2 Jilin Provincial Institute for Drug Control, Changchun 130033, China; 3 College of Pharmacy, Yanbian University, Yanji 133002, China; 4 College of Pharmacy, Beihua University, Jilin 132013, China

**Keywords:** *Gastrodia elata* extract, liver injury, D-galactose, PI3K-Akt signaling pathway, network pharmacology

## Abstract

In this research, we sought to methodically examine the protective effects of *Gastrodia elata* extract (GEE) on liver damage induced by D-galactose (D-gal) in mice and clarify the underlying mechanisms. The chemical composition of GEE was characterized using Ultra-Performance Liquid Chromatography–Tandem Mass Spectrometry (UPLC-MS/MS), while network pharmacology analysis was employed to predict potential molecular targets and signaling pathways. A mouse model of liver injury was established through daily intraperitoneal injection of D-gal over a 42-day period, during which the hepatoprotective efficacy of GEE was evaluated. Biochemical, histopathological, and molecular analyses were subsequently performed. UPLC-MS/MS identified ingredients such as amino acids, aromatic compounds, fatty acids, and terpenoids in GEE. A network pharmacology analysis enabled the identification of 272 common targets linked to GEE and liver damage, demonstrating notable enrichment within the phosphatidylinositol 3-kinase/protein kinase B (PI3K/Akt) signaling pathway. In vivo experiments demonstrated that GEE effectively alleviated D-gal-induced body weight loss and elevated liver index values, alleviated hepatic histological damage, and reduced serum levels of Alanine Aminotransferase (ALT), Aspartate Aminotransferase (AST), and Alkaline Phosphatase (ALP). Furthermore, GEE enhanced the activities of the antioxidant enzymes superoxide dismutase (SOD) and catalase (CAT), decreased malondialdehyde (MDA) levels, and downregulated the mRNA expression of the pro-inflammatory cytokines Interleukin-6 (*IL-6*), Interleukin-1 beta *(IL-1β*), and Tumor Necrosis Factor-alpha (*TNF-α*). Western blot analysis confirmed that GEE activated the PI3K/Akt pathway, as evidenced by increased ratios of phosphorylated Phosphatidylinositol 3-kinase/Phosphatidylinositol 3-kinase (p-PI3K/PI3K) and phosphorylated AKT/Protein Kinase B (p-AKT/AKT); restored the B-cell lymphoma 2-associated X protein/B-cell lymphoma-2 (Bax/Bcl-2) balance; and reduced cyclin-dependent kinase inhibitor 1 (p21) expression. The results suggest that GEE protects against D-gal-induced liver damage by reducing oxidative stress, inhibiting inflammatory responses, and modulating apoptosis through the activation of the PI3K/Akt signaling pathway, providing support for its potential use in hepatoprotection.

## 1. Introduction

Liver injury, characterized by dysfunction in detoxification and metabolism under pathological stress, remains a significant global health challenge [1,2]. Liver injuries are closely related to multiple pathogenic factors, oxidative stress, and inflammatory responses [3,4,5]. Liver damage and aging interact with each other: oxidative stress and inflammation accelerate liver damage, and a damaged liver in turn accelerates overall aging [6,7,8,9]. The D-gal-induced mouse model, which reliably recapitulates key features of liver injury and aging through mechanisms involving reactive oxygen species (ROS) and advanced glycation end products (AGEs), has been widely adopted in preclinical studies [10,11,12,13,14].

*Gastrodia elata*, a traditional medicinal herb, can calm liver Yang, alleviate wind, stop spasms, and unblock meridians [15,16]. Pharmacological studies show that the main active constituents of GEE—gastrodin and p-hydroxybenzyl alcohol—potently combine antioxidant, anti-inflammatory, and neuroprotective activities [17,18,19,20,21,22]. Current research suggests that GEE can positively impact drug-induced liver damage [23,24]. Nevertheless, there is a dearth of comprehensive studies addressing whether GEE offers protection against D-gal-induced liver injury, and there is a need to further elucidate its specific mechanisms of action.

In this study, we utilized a D-gal-induced mouse liver injury model for the first time to systematically evaluate the hepatoprotective effect of GEE. By integrating network pharmacology prediction with classical in vivo pharmacological experiments, we established a complete “prediction–validation” research framework. This approach not only elucidated and validated the underlying molecular mechanisms but also provided experimental support for the potential application of *Gastrodia elata* in the treatment of D-gal-induced liver injury.

## 2. Materials and Methods

### 2.1. Materials

*Gastrodia elata* was acquired from Beijing Tongrentang Co., Ltd., located in Beijing, China. Vitamin C was sourced from Beijing, Haiwang Center Pharmaceutical Co., Ltd., which is also based in Beijing, China. C57BL/6 mice, with a body weight ranging from 35 to 40 g, were obtained from Liaoning, Changsheng Bio-technology Co., Ltd., located in Benxi, China. D-(+)-galactose and polyvinylidene fluoride (PVDF) membranes were sourced from Wuhan Boster Biological Technology Co., Ltd., in Wuhan, China. MDA assay and CAT activity assay kits were both acquired from Wuhan Servicebio Technology Co., Ltd., which is also based in Wuhan, China. Additionally, various other kits, including the Amplex Red ALT Activity Assay Kit, Amplex Red AST Activity Assay Kit, Alkaline Phosphatase Assay Kit, enhanced chemiluminescence (ECL) kit, SDS-PAGE gel preparation kit, dual-color SDS-PAGE protein loading buffer (5×), skim milk powder, and the bicinchoninic acid (BCA) protein assay kit, were procured from Beyotime Biotechnology Co., Ltd., based in Shanghai, China. Furthermore, horseradish peroxidase (HRP)-conjugated antibodies, such as goat anti-rabbit IgG and goat anti-mouse IgG; RIPA lysis buffer (strong); a phosphatase inhibitor cocktail (100×); and various antibodies, including p21 (Batch No.: 10355-1-AP), Bax (Batch No.: BC014175), PI3K (Batch No.: BC030815), Bcl-2 (Batch No.: NM-009741), and AKT (Batch No.: BC000479), were all obtained from Wuhan Sanying Biotechnology Co., Ltd., in Wuhan, China. The phospho-AKT (p-AKT) antibody (Batch No.: AF0016) and phospho-PI3K (p-PI3K) antibody (Batch No.: AF3241) were sourced from Jiangsu Qinke Biological Research Center located in Liyang, China. Trizol was purchased from GeneBetter based in South San Francisco, CA, USA. All organic chemical reagents were obtained from Macklin Biochemical Technology Co., Ltd., in Shanghai, China.

### 2.2. Sample Preparation

The crushed *Gastrodia elata* powder was subjected to triple extraction using 95% ethanol as the solvent, maintaining a solid-to-liquid ratio of 10:1 (*v*/*w*). The extraction was performed under ultrasonic-assisted conditions (100 Hz) at 30 °C, with each extraction lasting 3 h. After each step, the mixtures were filtered. The filtrates obtained were merged, concentrated with a rotary evaporator, and ultimately freeze-dried to yield GEE.

### 2.3. UPLC-MS/MS Analysis

The chemical constituents of GEE were analyzed using Q-Orbitrap high-resolution liquid chromatography–mass spectrometry (UPLC-MS/MS). Specifically, the ultra-high-performance liquid chromatography (UHPLC) system employed was an UltiMate 3000 RS (Thermo Fisher Scientific, Shanghai, China), and the mass spectrometer was a Q Exactive high-resolution mass spectrometer (Thermo Fisher Scientific, China). In summary, an accurate weight of 0.1 g of the sample was combined with 500 μL of 80% methanol and grinding beads. The resultant mixture was ground for a duration of 5 min, then vortex-mixed for 10 min, and finally centrifuged at 13,000 rpm for another 10 min. The supernatant was then collected for further analysis. Chromatographic separation was performed on an AQ-C18 column (150 × 2.1 mm, 1.8 μm), employing a mobile phase that comprised 0.1% formic acid in water (referred to as solvent A) and methanol (designated as solvent B). A flow rate of 0.30 mL/min was established under gradient elution conditions (Table 1). The temperature of the column was kept constant at 35 °C, and the auto-sampler was maintained at 10 °C. A sample volume of 5 μL was applied. Detection via mass spectrometry was conducted using an electrospray ionization (ESI) source that was operated in a mode switching between positive and negative. Full MS scans were captured across the *m*/*z* range of 100–1500 with a resolution of 70,000, while data-dependent MS^2^ (dd-MS^2^) scans were recorded at a resolution of 17,500. The spray voltage was adjusted to 3.2 kV, and the capillary temperature was maintained at 300 °C. High-purity argon was utilized as the gas for collisions, while high-purity nitrogen served as both the sheath and auxiliary gas, with flow rates set at 40 L/min and 15 L/min, respectively. The heater for the auxiliary gas was adjusted to a temperature of 350 °C. Collision energies were administered at stepped intervals of 30, 40, and 60 eV. The total acquisition time was 30 min. Following preprocessing of the original data with Compound Discoverer 3.3, compounds were identified using the mzCloud database (https://www.mzcloud.org/ (accessed on 15 March 2025)). All solvents were sourced from Merck (Darmstadt, Germany), and ultra-pure water was produced using the Millipore system (Merck, Darmstadt, Germany).

### 2.4. Network Pharmacology Analysis

The potential targets of the active components in Gastrodia elata extract (GEE) were predicted using the SwissTargetPrediction (http://www.swisstargetprediction.ch/ (accessed on 7 March 2025)), SEA (https://sea.bkslab.org/ (accessed on 7 March 2025)), and PharmMapper databases (https://www.lilab-ecust.cn/pharmmapper/ (accessed on 7 March 2025)). The drug-likeness screening of these components was performed using the SwissADME (http://www.swissadme.ch/ (accessed on 7 March 2025)) platform based on high GI absorption and compliance with Lipinski’s and Veber’s rules. Targets related to “liver injury” were retrieved from the GeneCards (https://www.genecards.org/ (accessed on 7 March 2025)) and OMIM (https://www.omim.org/ (accessed on 7 March 2025)) databases. The subsequent standard network pharmacology workflow—including the identification of common targets, construction and topological analysis of the protein–protein interaction (PPI) network (via STRING, (https://string-db.org/ (accessed on 10 March 2025)), and screening of hub genes—was developed according to established methodologies. Gene Ontology (GO) and Kyoto Encyclopedia of Genes and Genomes (KEGG) pathway enrichment analyses were conducted using the “clusterProfiler” (version 4.12.0) R package.

### 2.5. Experimental Animals

This study was conducted in strict accordance with the ARRIVE Guidelines 2.0 and relevant national animal welfare regulations. Six-week-old male C57BL/6 mice were procured from Liaoning Changsheng Bio-technology Co., Ltd., in Benxi, China. All animal procedures were approved by the Animal Ethics Committee of Jilin Medical University (Approval Protocol Code: 2020-KJT056; Date of Approval: 3 September 2020). Following a week of adaptation to standard laboratory settings (with the temperature and humidity maintained at 22 ± 1 °C and 55 ± 5% and a 12 h light/dark cycle), the mice were divided randomly into five groups, each consisting of six individuals (*n* = 6 per group): a control group receiving 0.9% saline, a D-gal group treated with 800 mg/kg, a low-dose GEE group treated with 100 mg/kg, a high-dose GEE group treated with 200 mg/kg, and a VC group treated with 200 mg/kg. Except for the control group, all the other groups received daily intraperitoneal injections of D-gal (800 mg/kg) to induce aging. Simultaneously, every treatment group was subjected to the drug intervention through oral gavage for 42 continuous days. Upon completion of the experiment, all mice were anesthetized and subsequently euthanized. Serum and liver tissues were gathered for further biochemical and histological evaluations.

### 2.6. Hematoxylin and Eosin (HE) Staining of Liver Tissue

Specimens of liver tissue obtained from the mice were preserved in 4% paraformaldehyde for 12 h and then subjected to various processing procedures, such as washing, dehydration, clearing, infiltration with paraffin, embedding, and sectioning to a thickness of 3 to 5 μm. The sections subsequently underwent antigen retrieval, baking, HE staining, and mounting to obtain the final HE-stained liver tissue sections [25].

### 2.7. Liver Function Test

Measurements of serum levels of ALP, ALT, and AST were conducted using appropriate commercial kits, following the guidelines provided by the manufacturers. An Infinite E PLEX microplate reader (Tecan Group Ltd., Männedorf, Switzerland) was utilized to assess the absorbance of the samples, and the concentrations were derived from a standard curve. Data are derived from six independent biological replicates (*n* = 6).

### 2.8. Determination of SOD Activity, CAT Activity, and MDA Content in Liver Tissue

After the mice were anesthetized, their liver tissues were collected and processed according to the guidelines provided by the respective reagent kits to measure MDA levels and SOD and CAT activity. These measurements were performed using an Infinite E PLEX microplate reader (Tecan Group Ltd., Männedorf, Switzerland). Each sample was analyzed in three independent biological replicates (*n* = 3).

### 2.9. Determination of Reverse Transcription Quantitative Polymerase Chain Reaction (RT-qPCR)

Total RNA extraction was conducted by applying Trizol reagent to liver tissues from mice. Specifically, 1 mL of Trizol was mixed with 50–100 mg of tissue sample, and then thorough homogenization was carried out. Subsequently, 0.2 mL of chloroform was added, and the mixture was vigorously shaken for 15 s. The sample was then centrifuged at 12,000 rpm for 15 min at 4 °C to separate the upper aqueous phase. An equivalent volume of isopropanol was incorporated into this supernatant, and the mixture was allowed to sit at room temperature for 20–30 min to promote RNA precipitation. After this incubation period, centrifugation was performed at 7500 rpm for 10 min, and the RNA pellet was washed with 1 mL of 75% ethanol; this was followed by an additional centrifugation at 7500 rpm for 5 min. Finally, the purified RNA was resuspended in 30–100 μL of RNase-free deionized water (Biosharp, Beijing, China).

Complementary DNA (cDNA) was generated from the isolated RNA via a commercial kit for cDNA synthesis (Biosharp, China) utilizing a T100 Thermal Cycler (Bio-Rad Laboratories, Hercules, CA, USA). Gene expression was analyzed using SYBR Green Supermix (Bio-Rad, Shanghai, China) via a QuantStudio 3 Real-Time PCR System (Thermo Fisher Scientific, Waltham, MA, USA). The relative mRNA expression levels were calculated using the 2^−ΔΔCt^ method. Table 2 includes the sequences of the primers utilized.

### 2.10. Western Blot

The liver tissues were subjected to complete protein extraction using an RIPA lysis buffer that included protease and phosphatase inhibitors. The concentration of protein was quantified with a BCA assay kit. Next, the protein samples were combined with a 4× loading buffer in a 1:4 ratio and heated at 100 °C for 10 min to denature the proteins. Afterwards, the proteins were separated via SDS-PAGE at a voltage of 80 V and transferred to PVDF membranes at 240 mA using a PowerPac™ Basic Electrophoresis Apparatus (Bio-Rad Laboratories, Hercules, CA, USA). The membranes were then incubated with 5% skim milk for 2 h at room temperature and subsequently washed with TBST. The membranes were then incubated overnight at 4 °C with the following primary antibodies: β-actin (1:5000), Bax (1:5000), p21 (1:2000), Bcl-2 (1:3000), p-PI3K (1:1000), PI3K (1:5000), p-AKT (1:1000), and AKT (1:3000). After a rinse with TBST, the membranes were treated with species-specific secondary antibodies for 2 h at room temperature. Protein bands were detected using an enhanced chemiluminescence (ECL) system and imaged with an ImageQuant 800 system (Cytiva, Marlborough, MA, USA). Band intensities were quantified using the ImageJ software (version 1.53, National Institutes of Health, Bethesda, MD, USA).

### 2.11. Statistical Analysis

The results are expressed as the mean ± standard deviation (SD) from six independent biological replicates (*n* = 6). Statistical evaluations were performed using the GraphPad Prism software (version 9.5). The normality of all datasets was confirmed using the Shapiro–Wilk test, and the homogeneity of variances was verified using Levene’s test. One-way analysis of variance (ANOVA) was utilized to examine multiple groups. In cases where a significant overall effect was identified (*p* < 0.05), Dunnett’s post hoc test was applied for targeted comparisons with the control group. A *p*-value below 0.05 was considered statistically significant.

## 3. Results

### 3.1. Chemical Constituents of GEE Were Analyzed via UPLC-MS/MS

The GEE was characterized using UPLC-MS/MS by combining precise quality measurements, MS/MS fragment mode, comparison with reference standards, published research data, and the HMDB, METLIN, and mzCloud databases. A total of 18 components were identified or preliminarily identified in GEE. These components are classified into six major categories (Figure 1A): aromatics (six compounds, 33.33%), amino acids (five compounds, 27.77%), fatty acids (three compounds, 16.67%), terpenes (two compounds, 11.11%), coumarins (one compound, 5.56%), and other (one compound, 5.56%) (Figures S1–S14, Table S2).

### 3.2. Acquisition and Screening of Potential Targets of GEE for Liver Injury

**Figure 1:**
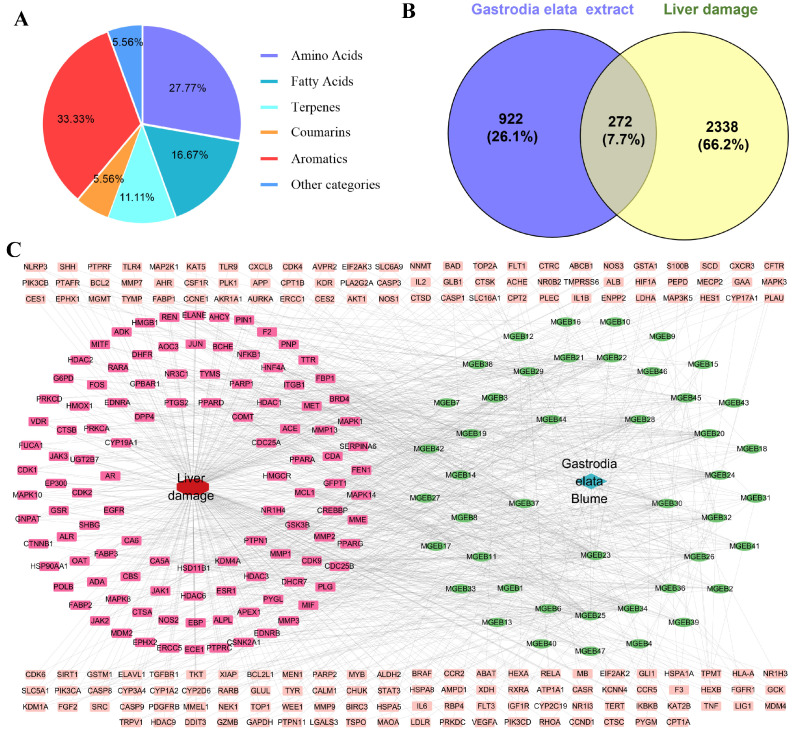
The chemical constituents of GEE, target collection, and prediction results: (**A**) classification of the compounds contained in GEE using UPLC-MS/MS; (**B**) Venn diagram of the target of *Gastrodia elata* extract in treating liver injury; and (**C**) drug–disease–target network visualization.

Through screening and deduplication from the SEA, PharmMapper, and SwissTarget Prediction databases, we identified a total of 1194 potential targets for GEE. Additionally, 2610 targets associated with liver injury were gathered from the GeneCards and OMIM databases. An analysis of the relationship between targets associated with GEE and those linked to liver injury revealed 272 shared targets (Figure 1B), identified as potential therapeutic targets for GEE in the mitigation of liver injury. These 272 shared targets were then uploaded to Cytoscape 3.7.0 to create a “Compound-Target-Disease” network. The resulting network consisted of 319 nodes and 1091 edges, with each edge representing an interaction between either a compound and a target or a target and the disease (Figure 1C).

### 3.3. PPI Network Construction and Core Target Screening

**Figure 2:**
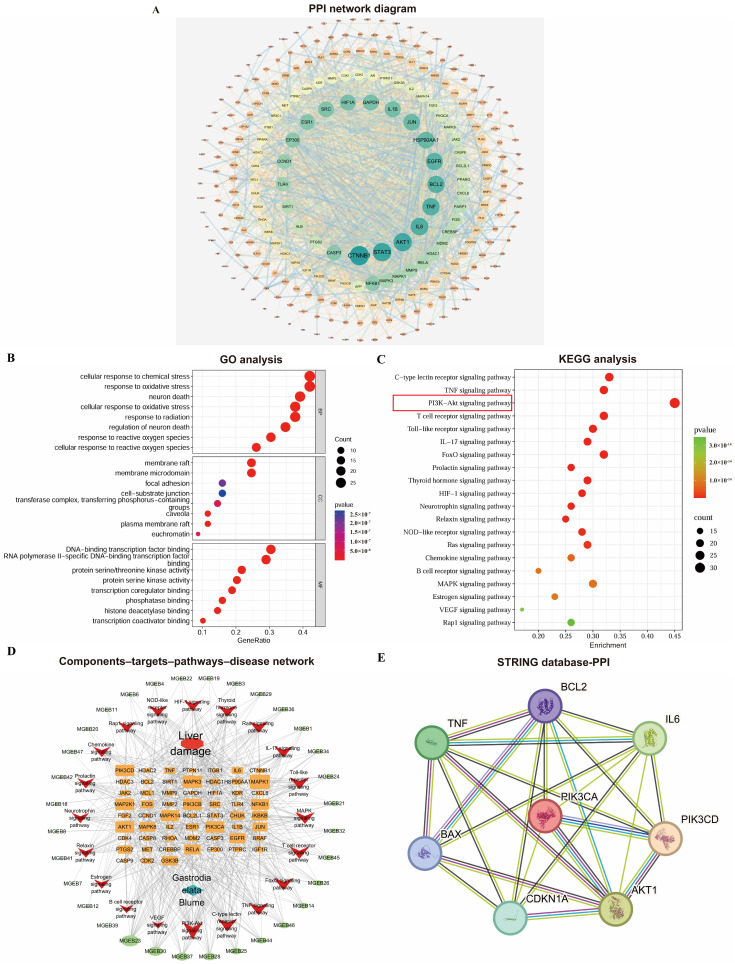
Comprehensive analysis of network pharmacology: (**A**) PPI network diagram of *Gastrodia elata* targets for liver injury treatment; (**B**) bubble plots of GO analyses; (**C**) bubble plots of KEGG enrichment analyses; (**D**) *Gastrodia elata* extract components–targets–pathways–disease network visualization. (**E**) Protein interaction analysis diagram based on STRING database.

The PPI network was constructed using the STRING database and Cytoscape 3.7.0 (Figure 2A). It consisted of 260 nodes and 2002 edges, where nodes represent proteins and edges represent interactions. Node size and color (ranging from orange to green) reflect their degree values, while edge thickness corresponds to the combined score strength. Targets with a degree value greater than twice the median (i.e., ≥18) were selected as core targets. These were further screened based on betweenness centrality (>0.0177) and closeness centrality (>0.4451) to identify key targets.

### 3.4. GO and KEGG Pathway Enrichment Analysis

The GO enrichment analysis yielded a total of 2822 significant terms (*p* < 0.05), including 2546, 107, and 168 in the BP, CC, and MF categories. The top 10 significantly enriched terms from each category were selected based on their *p*-values and visualized in a bubble plot (Figure 2B). Based on the KEGG pathway enrichment analysis, a total of 171 pathways significantly associated with the core targets were obtained (*p* < 0.05). Figure 2C displays the top 20 pathways ranked by *p*-value; the PI3K-Akt signaling pathway showed extremely high enrichment significance and its Enrichment score was the largest (Enrichment score = 0.45). This initially demonstrated from the perspective of bioinformatics that this pathway plays a key role in the process of GEE intervention in D-gal liver injury.

### 3.5. Construction of Active Ingredient–Intersection Target–Pathway Network

A target gene–pathway interaction network was constructed using Cytoscape 3.7.0 (Figure 2D). Subsequently, through the analysis of the STING database, it was found that there were interactions among proteins such as PI3K/AKT, IL-6, TNF, IL-1β, p21, BAX, and BCL2. This pathological mechanism, target enrichment biological process, and the function of the candidate core pathway were highly consistent (Figure 2E). It provides the strongest biological rationality for us to focus on the PI3K/Akt pathway.

### 3.6. Evaluation of Mouse Body Weight and Liver Indices

**Figure 3:**
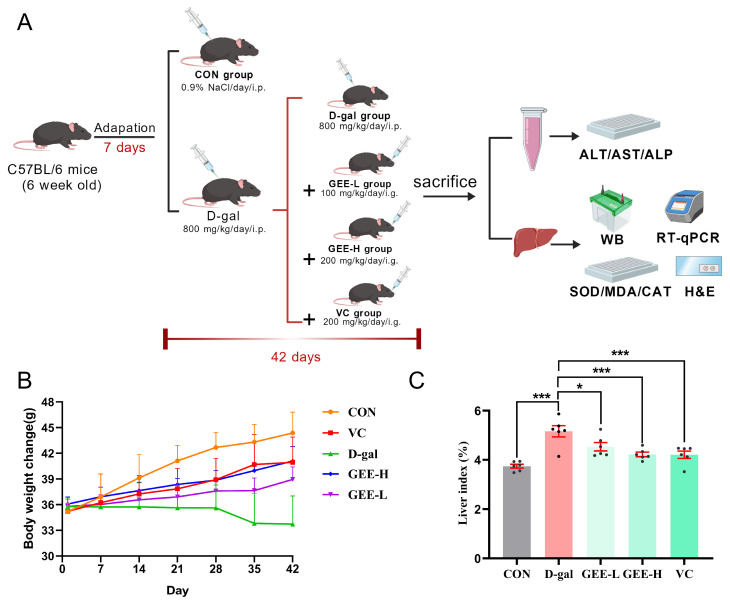
Assessment of GEE’s protective role with respect to liver damage induced by D-gal. (**A**) Diagrammatic representation of the experimental setup involving mice. (**B**) Changes in body weight were recorded (*n* = 6). (**C**) The liver index was evaluated (*n* = 6). Statistical analysis was performed using one-way ANOVA, with subsequent application of Dunnett’s post hoc test. Differences were considered statistically significant at * *p* < 0.05, and *** *p* < 0.001 relative to the D-gal group (*n* = 6, Mean ± SD).

To further evaluate the hepatoprotective effect of GEE against D-gal-induced liver injury, we established a mouse model and monitored relevant physiological parameters (Figure 3A). Changes in body weight and liver index values are recognized as important indicators for assessing liver damage in mice. As illustrated in (Figure 3B,C), compared with the control group, the D-gal group exhibited a significant decrease in body weight and a marked increase in liver index values (*p* < 0.001). However, treatment with GEE significantly reversed these alterations in a dose-dependent manner, with the high-dose group (*p* = 0.0007) showing a more pronounced effect than the low-dose group (*p* = 0.0173). These results preliminarily demonstrate that GEE can alleviate D-gal-induced liver injury in mice.

### 3.7. Hepatic Histopathological Analysis

**Figure 4:**
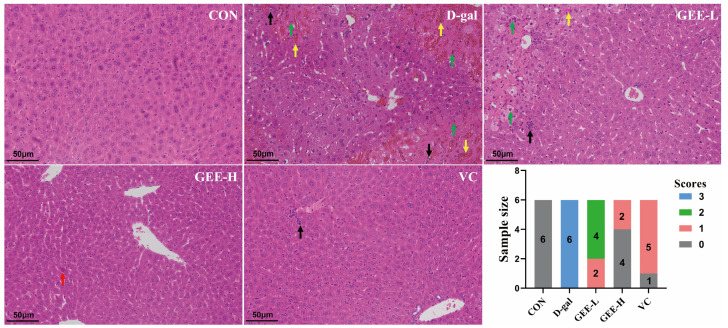
Evaluation of liver tissue morphology by H&E staining (200× magnification). Score 0 indicates normal liver tissue; Score 1 indicates only a small amount of liver cell degeneration and necrosis as well as inflammatory infiltration; Score 2 indicates that the liver lobule structure is still intact, some liver cells are degenerated and necrotic, and the liver tissue shows a lot of inflammatory cell infiltration; Score 3 indicates that congestion occurs in the hepatic sinusoids and central veins, extensive liver cell degeneration and necrosis occur, the liver tissue shows a large amount of inflammatory cell infiltration, and some liver lobule structures are damaged. The green arrow indicates severe architectural disruption of hepatic tissue accompanied by patchy necrosis of hepatocytes. The yellow arrow points to hepatic congestion and disarray in the arrangement of hepatic cords. The black arrow marks a mild degree of inflammatory cell infiltration. The red arrow highlights a mild structural abnormality in the liver tissue.

To evaluate the hepatoprotective effect of GEE with respect to D-gal-induced injury, we performed histological analysis of liver tissues via H&E staining (Figure S23, Table S1). As shown in Figure 4, liver sections from the control group exhibited an intact hepatic architecture with abundant cytoplasm, clear nuclear outlines, and orderly arranged hepatic cords, without significant inflammatory cell infiltration. In contrast, liver tissues from the D-gal group displayed marked pathological alterations, including hepatocellular edema, necrosis with nuclear pyknosis and karyorrhexis (indicated by black arrows), cytoplasmic vacuolization, and inflammatory cell infiltration. However, the histopathological changes induced by D-gal were significantly attenuated by GEE treatment. Specifically, GEE administration reduced hepatocellular necrosis, nuclear fragmentation, and inflammatory infiltration. Notably, the effect was more pronounced in the high-dose GEE group than in the low-dose group. These findings demonstrate that GEE effectively alleviates D-gal-induced hepatic histopathological damage.

### 3.8. Effects of GEE on Serum Enzymes

**Figure 5:**
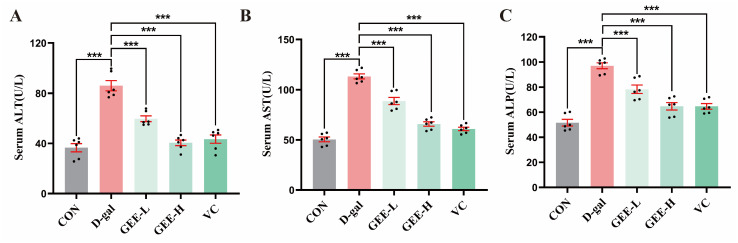
Liver function indicator levels: (**A**) serum ALT, (**B**) AST, and (**C**) ALP levels in mice. Statistical analysis was performed using one-way ANOVA, with subsequent application of Dunnett’s post hoc test. Differences were considered statistically significant at *** *p* < 0.001 relative to the D-gal group (*n* = 6, Mean ± SD).

Serum levels of ALT, AST, and ALP are key biochemical indicators useful for assessing the extent of hepatocyte damage. These enzymes are primarily located within hepatocytes. When liver injury occurs, increased permeability of the hepatocyte membrane leads to the release of ALP, ALT, and AST into the bloodstream, resulting in elevated serum levels of these enzymes [26]. As shown in Figure 5A–C, compared with the control group, the levels of ALP (*p* < 0.001), ALT (*p* < 0.001), and AST (*p* < 0.001) in the serum of mice in the D-gal group were significantly higher, indicating successful construction of the liver injury model. Treatment with GEE significantly attenuated these elevations in a dose-dependent manner. Specifically, the low- and high-dose GEE groups had reduced ALT (all *p* < 0.001), AST (all *p* < 0.001), and ALP levels (*p* = 0.0003 and *p* < 0.001). Among them, the intervention effect observed for the high-dose GEE group was similar to that observed for the VC group, suggesting that GEE has a significant protective effect with respect to liver injury.

### 3.9. Measurement of Antioxidant Enzyme Activity in Liver Tissue

**Figure 6:**
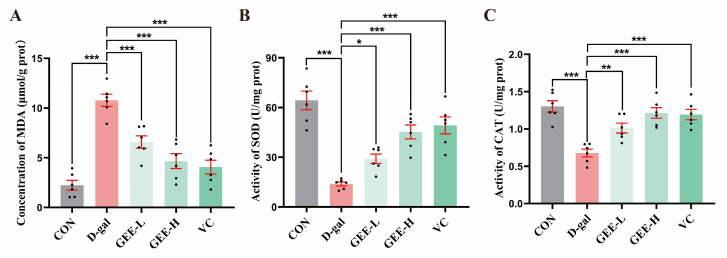
Levels of antioxidant enzyme activity in liver tissue: (**A**) concentration of MDA; (**B**) SOD activity; and (**C**) CAT activity. Statistical analysis was performed using one-way ANOVA, with subsequent application of Dunnett’s post hoc test. Differences were considered statistically significant at * *p* < 0.05, ** *p* < 0.01, and *** *p* < 0.001 relative to the D-gal group (*n* = 6, Mean ± SD).

Assessment of oxidative stress in liver tissues revealed that, compared with the D-gal group, all the GEE-treated groups and the VC positive control group exhibited significant improvements in antioxidant capacity (Figure 6A–C). Specifically, the GEE intervention significantly elevated SOD activity (GEE-L: *p* = 0.0481; GEE-H: *p* < 0.001) and CAT activity (GEE-L: *p* = 0.0065; GEE-H: *p* < 0.001) while effectively reducing MDA content (GEE-L: *p* = 0.0004; GEE-H: *p* < 0.001). These results suggest that GEE may alleviate oxidative stress and exert hepatoprotective effects by enhancing SOD and CAT activity while reducing MDA accumulation.

### 3.10. GEE Attenuates D-Gal-Induced Hepatic Pro-Inflammatory Cytokine mRNA Expression in Mice

**Figure 7:**
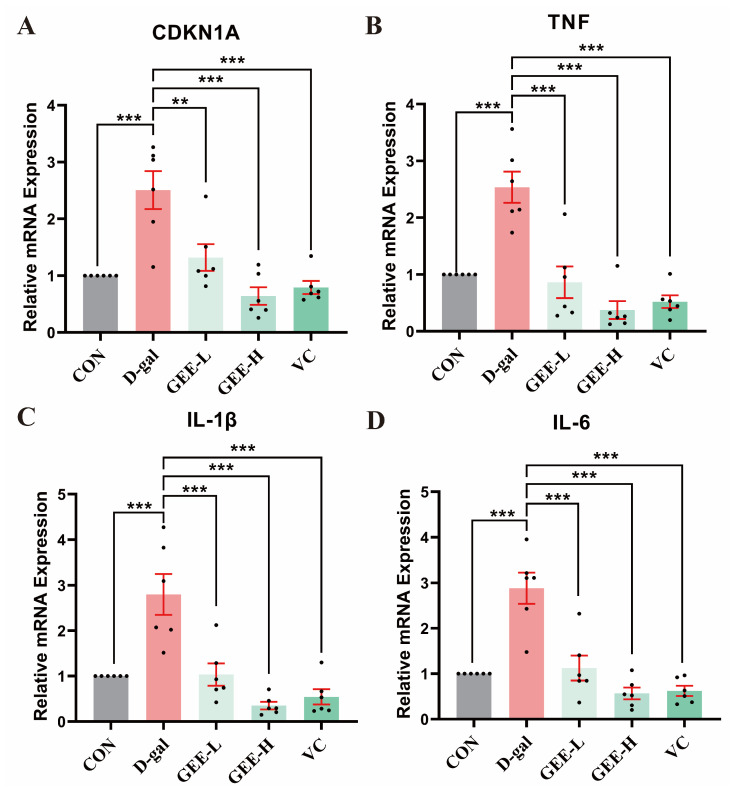
RT-qPCR analysis was conducted to assess the expression levels of genes in liver tissue: levels of (**A**) *CDKN1A*, (**B**) *TNF*, (**C**) *IL-1β*, and (**D**) *IL-6*. Statistical analysis was performed using one-way ANOVA, with subsequent application of Dunnett’s post hoc test. Differences were considered statistically significant at ** *p* < 0.01, and *** *p* < 0.001 relative to the D-gal group (*n* = 6, Mean ± SD).

To identify the ameliorative effect of GEE on D-gal-induced chronic inflammation, we assessed the mRNA expression levels of pro-inflammatory cytokines (*IL-6*, *IL-1β*, and *TNF-α*) and the senescence-associated gene *CDKN1A* in mouse liver tissues. As shown in Figure 7, relative to the control group, the mRNA expression levels of these genes were significantly elevated in the D-gal model group (*CDKN1A*: *p* = 0.0002; *TNF-α*: *p* < 0.001; *IL-1β*: *p* = 0.0002; *IL-6*: *p* < 0.001). In contrast, GEE administration effectively inhibited this upregulation, with effects that were comparable to those of the VC positive control. Notably, the high-dose group (*CDKN1A*, *TNF-α*, *IL-1β*, *IL-6*: all *p* < 0.001) exhibited a more pronounced downregulation of gene expression than the low-dose group (*CDKN1A*: *p* = 0.0029; *TNF-α*: *p* < 0.001; *IL-1β*: *p* = 0.0003; *IL-6*: *p* < 0.001), indicating that GEE exerts dose-dependent anti-inflammatory and anti-aging effects.

### 3.11. Western Blot Analysis of the Protective Effects of GEE on Liver Injury via the PI3K/Akt Signaling Pathway

**Figure 8:**
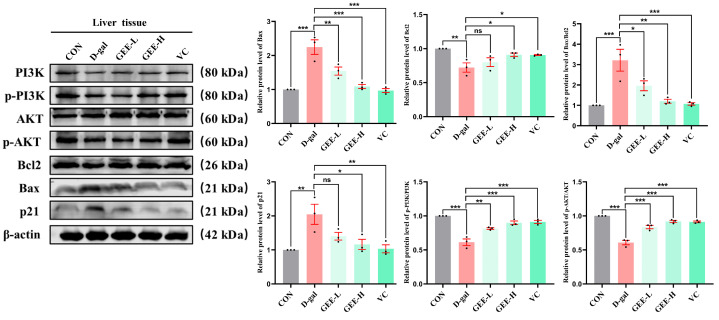
The level protein expression of Bcl2, Bax, p-PI3K, p-AKT, and p21 in liver tissue, as determined via Western blot. Statistical analysis was performed using one-way ANOVA, with subsequent application of Dunnett’s post hoc test. Differences were considered statistically significant at * *p* < 0.05, ** *p* < 0.01, and *** *p* < 0.001 relative to the D-gal group (*n* = 3, Mean ± SD).

To further investigate the molecular mechanism underlying GEE-mediated alleviation of D-gal-induced chronic liver inflammation, we performed immunoblotting to analyze protein expression levels in liver tissues. The results demonstrated that D-gal-induced liver injury significantly inhibited the PI3K/AKT signaling pathway, as evidenced by reduced p-PI3K/PI3K (*p* < 0.001) and p-AKT/AKT (*p* < 0.001) ratios. Apoptotic balance was also disrupted, with upregulation of Bax (*p* < 0.001), downregulation of Bcl-2 (*p* = 0.0037), and elevated p21 expression (*p* = 0.0039), indicating cell cycle arrest and senescence. Treatment with GEE markedly attenuated these alterations by activating the PI3K/AKT pathway, as shown by increased p-PI3K/PI3K (GEE-L: *p* = 0.0013; GEE-H: *p* < 0.001) and p-AKT/AKT (all *p* < 0.001) ratios. Furthermore, GEE restored apoptotic equilibrium by downregulating Bax (GEE-L: *p* = 0.0051; GEE-H: *p* = 0.0001) and upregulating Bcl-2 (GEE-H: *p* = 0.0376) while reducing p21 expression (GEE-H: *p* = 0.0114). Collectively, these results indicate that GEE mitigates D-gal-induced liver injury by promoting PI3K/AKT signaling, reestablishing apoptotic homeostasis, and alleviating p21-associated senescence (Figure 8 and Figures S15–S22).

## 4. Discussion

The liver, as a central metabolic organ, is highly susceptible to damage from various factors, with oxidative stress and inflammatory responses playing pivotal roles in the pathogenesis of liver injury [27,28,29,30]. The aging process further exacerbates this susceptibility, creating a vicious cycle that accelerates hepatic dysfunction. In this study, we observed that GEE exerts a significant protective effect against D-gal-induced liver injury in mice. Correlative changes were noted between this protective effect and the activation of the PI3K/Akt signaling pathway, reduction in oxidative stress, inhibition of inflammation, and attenuation of cellular senescence.

The chemical composition of GEE was first characterized using UPLC-MS/MS, through which 18 major constituents were identified, including amino acids, aromatic compounds, fatty acids, and terpenoids [31,32,33]. These bioactive components may serve as the material basis for the biological activities observed in this study. Furthermore, network pharmacological analysis was employed to systematically predict potential mechanisms of action, revealing that the predicted therapeutic targets of GEE in liver injury are significantly enriched in pathways related to oxidative stress, apoptosis, and inflammation, with the PI3K/Akt signaling pathway among the most prominent. This bioinformatics-based prediction provided a theoretical framework for subsequent experimental validation.

The success of the D-gal-induced liver injury model was confirmed by significant changes in physiological and biochemical indicators. Weight loss and increased liver indices in the model group are typical signs of metabolic disorders and organ damage [34]. Histopathological observations provided direct evidence of liver injury, including hepatocyte edema, necrosis, and inflammatory infiltration [35]. GEE treatment effectively reversed these pathological changes and restored liver structure to near-normal in a dose-dependent manner. This morphological improvement was accompanied by the recovery of serum liver enzyme (ALT, AST, ALP) levels. Elevated levels of these enzymes are a direct marker of impaired liver cell membrane integrity [36], and GEE significantly inhibited this elevation, indicating its ability to stabilize liver cell membranes and reduce cell damage.

A key finding of this study is the antioxidant activity of GEE observed in the D-gal-induced model. D-gal-induced liver injury is primarily mediated by oxidative stress through the generation of ROS and AGEs [37]. Our results showed that GEE enhanced the activities of critical antioxidant enzymes (SOD and CAT) while reducing the level of MDA, a marker of lipid peroxidation. By augmenting the endogenous antioxidant defense system, GEE may mitigate oxidative damage to cellular macromolecules (particularly lipids), which could contribute to protecting hepatocytes from apoptosis and necrosis.

GEE also exhibited anti-inflammatory effects in this model. D-gal induction led to upregulated mRNA expression of pro-inflammatory cytokines (*IL-6*, *IL-1β*, and *TNF-α*), which are core drivers of the inflammatory cascade in persistent liver injury [38]. GEE treatment significantly inhibited the expression of these cytokines, indicating suppression of the inflammatory response. The combination of antioxidant and anti-inflammatory effects may create a favorable environment for liver repair and regeneration.

**Figure 9:**
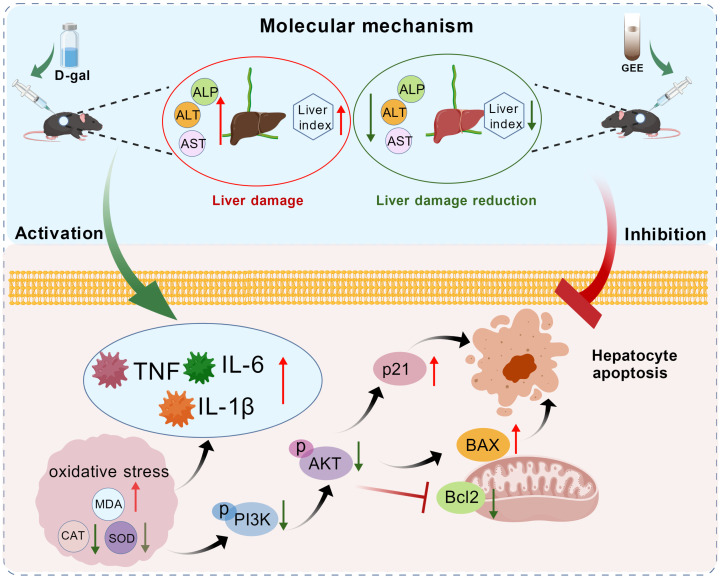
The protective mechanism of GEE with respect to D-gal-induced liver injury in mice via the PI3K/Akt signaling pathway.

Notably, our research investigated the molecular mechanisms underlying GEE’s protection and found that the PI3K/Akt pathway is involved in this process (Figure 9). The PI3K/Akt pathway is a key regulator of cell survival, proliferation, metabolism, and apoptosis [39]. Western blotting analysis showed that D-gal administration inhibited the PI3K/Akt pathway, as indicated by decreased levels of p-PI3K and p-Akt. This inhibition was associated with dysregulated apoptosis (increased Bax/Bcl-2 ratio) and induced cellular senescence (elevated p21 expression). GEE treatment effectively activated the PI3K/Akt pathway, rebalanced the Bax/Bcl-2 ratio, and reduced p21 expression. Activation of PI3K/Akt by GEE may promote cell survival by inhibiting pro-apoptotic signals, and this pathway may also be linked to the observed reductions in oxidative stress and inflammation. These findings are consistent with our network pharmacology predictions and provide experimental support for the involvement of the PI3K/Akt pathway in GEE’s hepatoprotective effect.

Despite the meaningful findings, this study has limitations that should be acknowledged. We only identified the main chemical components of GEE through UPLC-MS/MS, but did not conduct quantitative analysis on these components. Aromatic compounds, amino acids and terpenoids have been confirmed to be the main bioactive components, but their exact contents in the extracts remain unclear. This lack of quantification hinders the establishment of a clear structure–activity relationship between specific components of GEE and their liver-protective effects, thus making it impossible to determine which components play a leading role in activating the PI3K/Akt pathway or regulating oxidative stress and inflammation. To address these limitations, future research will prioritize the quantitative determination of key bioactive components in GEE using the validated UPLC-MS/MS method. Then, we will evaluate the individual and synergistic effects of these quantitative components in in vitro hepatocyte models and various in vivo liver injury models to clarify their respective contributions.

## 5. Conclusions

This research illustrates that GEE has a notable protective impact against liver damage caused by D-gal in mice. The mechanism by which GEE exerts its hepatoprotective effects is primarily facilitated by the activation of the PI3K/Akt signaling pathway. This activation leads to a reduction in oxidative stress (increased SOD and CAT levels and decreased MDA levels), inhibition of inflammation (downregulated IL-6, IL-1β, and TNF-α), restoration of apoptotic balance (modulated Bax/Bcl-2), and attenuation of cellular senescence (reduced p21 levels). Consequently, GEE treatment improved liver histopathology and normalized serum levels of liver enzymes (ALT, AST, and ALP). These findings provide a solid scientific basis for the application of *Gastrodia elata* in alleviating liver injury and suggest its potential value in developing therapeutic strategies for oxidative-stress- and aging-related liver diseases.

## Figures and Tables

**Table 1:** Chromatographic Gradient.

Time (min)	A (%)	B (%)
1	98	2
5	80	20
10	50	50
15	20	80
20	5	95
27	5	95
28	98	2
30	98	2

**Table 2:** Primers used in the study.

Genes	Primers	Sequence 5e > 3e
*IL-6*	Forward	gccttcttgggactgatgct
	Reverse	agcctccgacttgtgaagtg
*IL-1β*	Forward	gcaactgttcctgaactcaact
	Reverse	atcttttggggtccgtcaact
*TNF*	Forward	ggactagccaggagggagaa
	Reverse	cgcggatcatgctttctgtg
*CDKN1A*	Forward	cagctcagtggactggaagg
	Reverse	tagaatgctctgggaggcct

## Data Availability

The original contributions presented in the study are included in the article/Supplementary Materials. Further inquiries can be directed to the corresponding author.

## Supplementary Materials

The following supporting information can be downloaded at https://www.mdpi.com/article/10.3390/cimb48010006/s1.
