# Supplementary material for: Exploring the Protective Effect of Gastrodia elata Extract on D-Galactose-Induced Liver Injury in Mice Based on the PI3K/Akt Signaling Pathway
Source: Curr Issues Mol Biol. 2025 Dec 20;48(1):6. doi: 10.3390/cimb48010006 (PMC12840178; doi:10.3390/cimb48010006)
Supplement: Supplementary file 1 [file cimb-48-00006-s001.zip › cimb-4034100-supplementary.pdf]

Exploring the Protective Effect of *Gastrodia elata* Extract on D-Galactose-Induced Liver Injury in Mice Based on the PI3K/Akt Signaling Pathway

Liu Han [1,†], Hongyu Zhai [2,†], Xiangyu Ma [1,3], Qiaosen Ren [2], Jiating Liu[1], Zhe Zhang[1], Xintong Li[1], Qiuyue Zhang [1,*]and Xin Sun [1,*]

[1] College of Pharmacy, Jilin Medical University, Jilin 132013, China
[2] Jilin Provincial Institute for Drug Contro, Changchun 130033, China
[3] College of Pharmacy, Yanbian University, Yanji 133002, China
* Correspondence: zqy240412@jlmu.edu.cn (Q.Z.); sunxin@jlmu.edu.cn (X.S.)
† These authors contributed equally to this work.

Figure S1. The total ion chromatogram of GEE in positive and negative ion modes.
Figure S2. The mass spectrum of L-Isoleucine.
Figure S3. The mass spectrum of L-Tyrosine.
Figure S4. The mass spectrum of 5-Hydroxymethyl-2-furaldehyde.
Figure S5. The mass spectrum of Stearic acid.
Figure S6. The mass spectrum of Oleic acid.
Figure S7. The mass spectrum of Linoleic acid.
Figure S8. The mass spectrum of D-(-)-Aspartic acid.
Figure S9. The mass spectrum of L-Valine.
Figure S10. The mass spectrum of (-)-Caryophyllene oxide.
Figure S11. The mass spectrum of Vanillin.
Figure S12. The mass spectrum of Esculetin.
Figure S13. The mass spectrum of 3-(4-hydroxy-3-methoxyphenyl)propanoic acid.
Figure S14. The mass spectrum of 3-Hydroxybenzoic acid.
Figure S15. The original Western blot analysis image of p-PI3K protein.
Figure S16. The original Western blot analysis image of PI3K protein.
Figure S17. The original Western blot analysis image of p-AKT protein.
Figure S18. The original Western blot analysis image of p21 protein.
FigureS19. The original Western blot analysis image of Bcl2 protein.
Figure S20. The original Western blot analysis image of Bcl2 protein.
Figure S21. The original Western blot analysis image of AKT protein.
Figure S22. The original Western blot analysis image of β-actin protein.
Figure S23. The original images of liver tissue morphology by H&E staining.
Table S1. The score results of HE staining.
Table S2. The full component identification form of GEE.

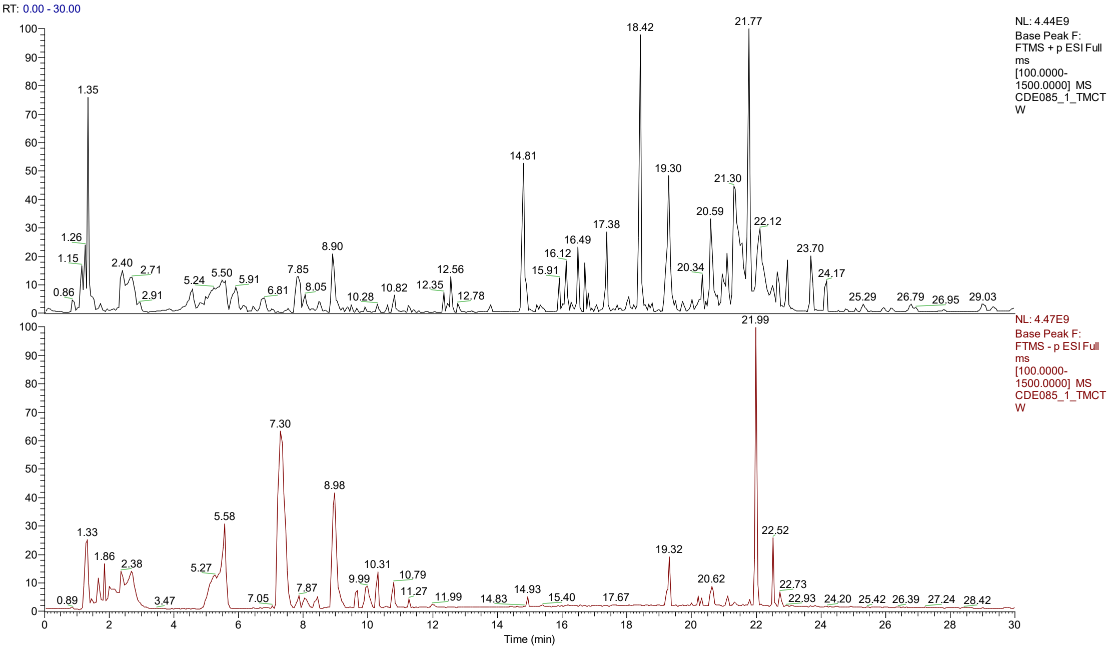

Figure S1. The total ion chromatogram of GEE in positive and negative ion modes.

Structure	Name	RT [min]	Formula	Calc. MW	mzCloud Best Match
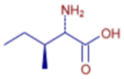	L-Isoleucine	1.75	C6 H13 N O2	131.09459	99.6992389129005

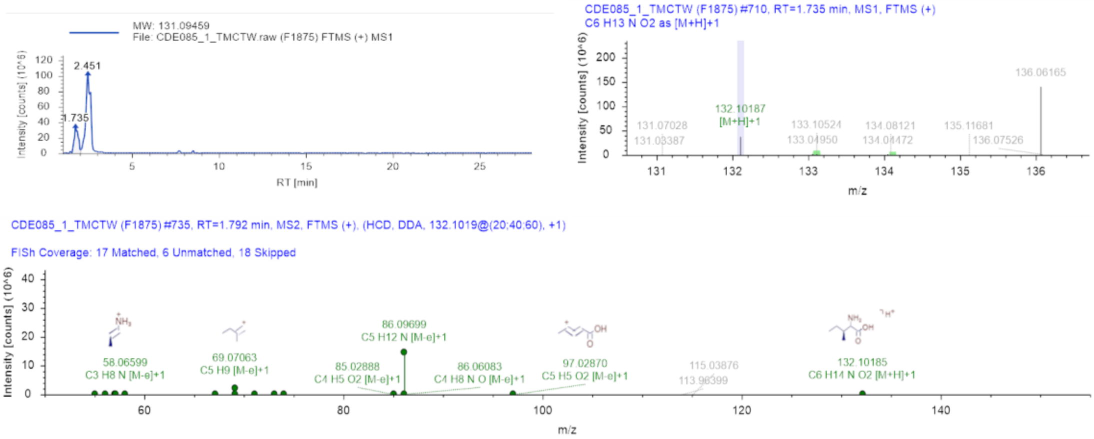

Figure S2. The mass spectrum of L-Isoleucine.

Structure	Name	RT [min]	Formula	Calc. MW	mzCloud Best Match
	L-Tyrosine	1.51	C9 H11 N O3	164.04717	99.6848935432966

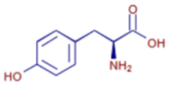

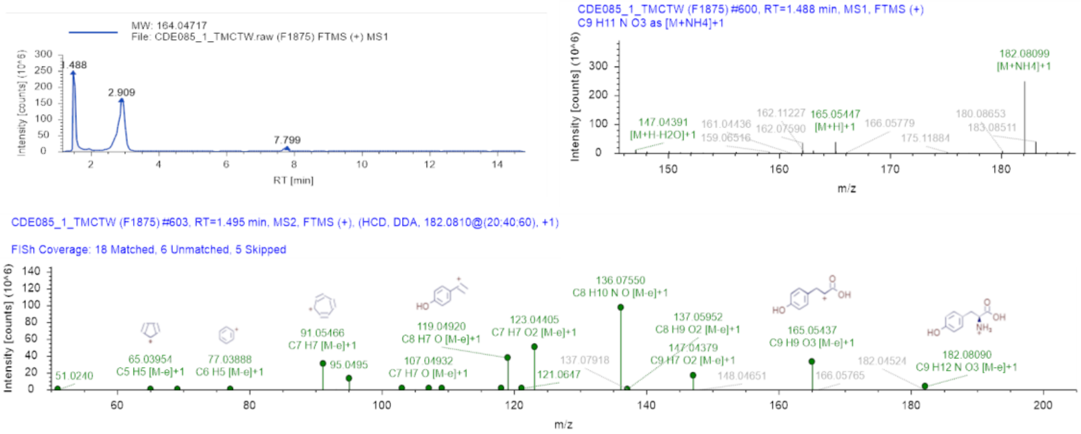

Figure S3. The mass spectrum of L-Tyrosine.

Structure	Name	RT [min]	Formula	Calc. MW	mzCloud Best Match
	5-Hydroxymethyl-2-furaldehyde	5.91	C6 H6 O3	126.03177	99.6098688344697

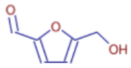

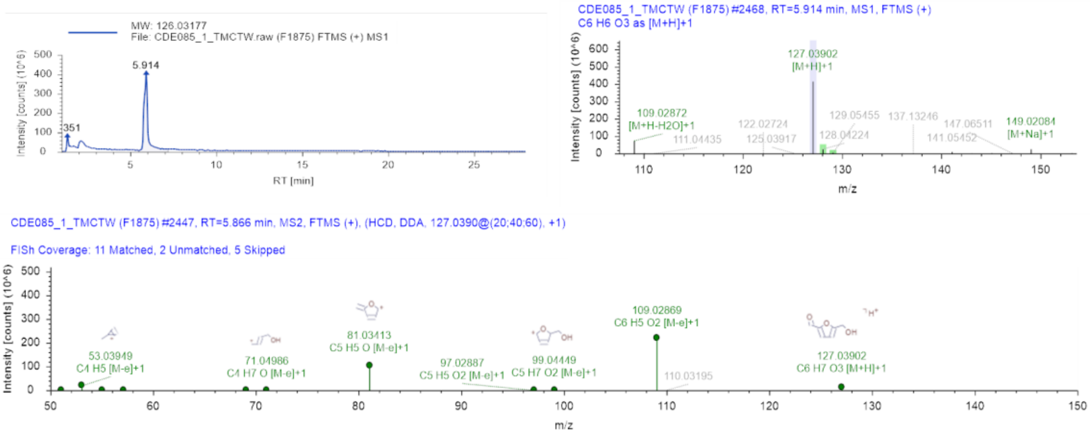

Figure S4. The mass spectrum of 5-Hydroxymethyl-2-furaldehyde.

Structure	Name	RT [min]	Formula	Calc. MW	mzCloud Best Match
	Stearic acid	23.83	C18 H36 O2	284.27144	**99.1005905097238**

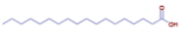

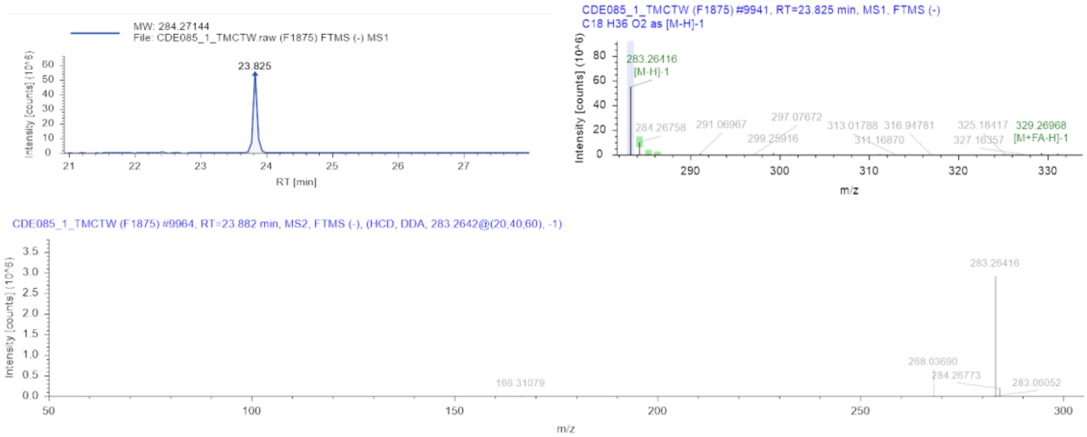

Figure S5. The mass spectrum of Stearic acid.

Structure	Name	RT [min]	Formula	Calc. MW	mzCloud Best Match
	Oleic acid	22.74	C18 H34 O2	282.25546	**99.0691387889269**

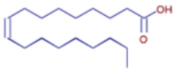

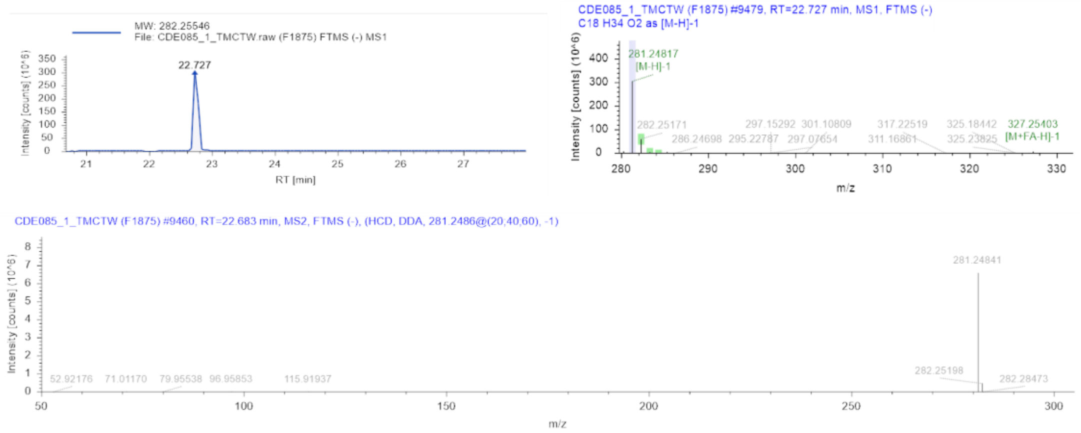

Figure S6. The mass spectrum of Oleic acid.

Structure	Name	RT [min]	Formula	Calc. MW	mzCloud Best Match
	Linoleic acid	20.81	C18 H32 O2	280.24006	**99.0418871860567**

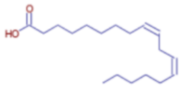

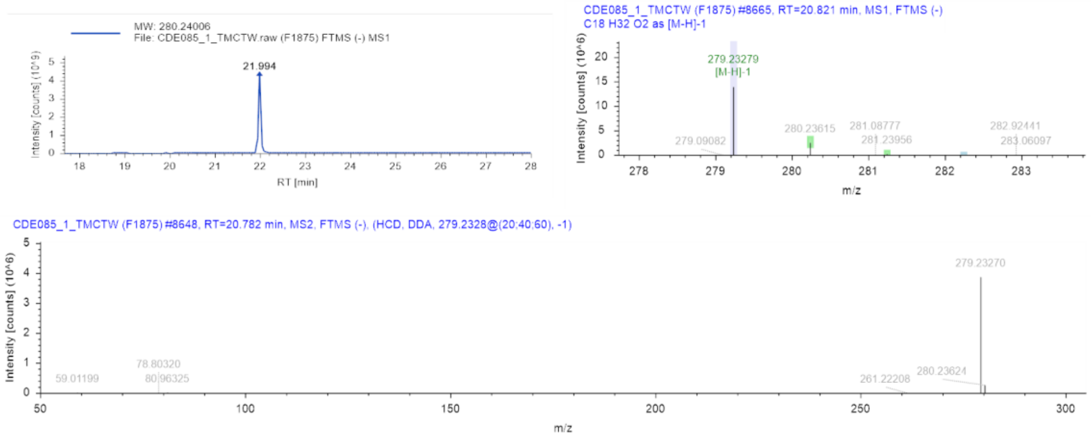

Figure S7. The mass spectrum of Linoleic acid.

Structure	Name	RT [min]	Formula	Calc. MW	mzCloud Best Match
	D-(-)-Aspartic acid	1.27	C4 H7 N O4	133.03731	**98.9453143054193**

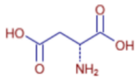

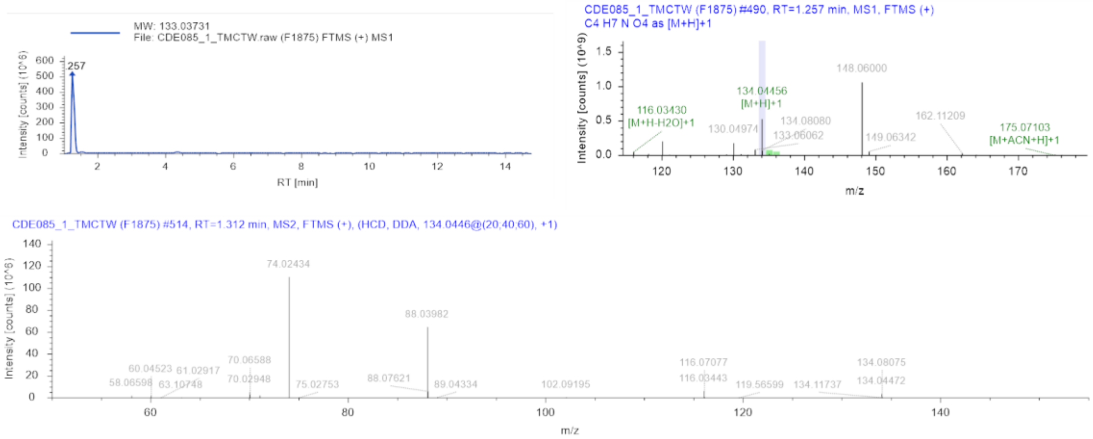

Figure S8. The mass spectrum of D-(-)-Aspartic acid.

Structure	Name	RT [min]	Formula	Calc. MW	mzCloud Best Match
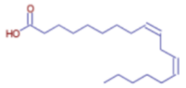	Linoleic acid	22.47	C18 H32 O2	280.24008	98.9170398140642

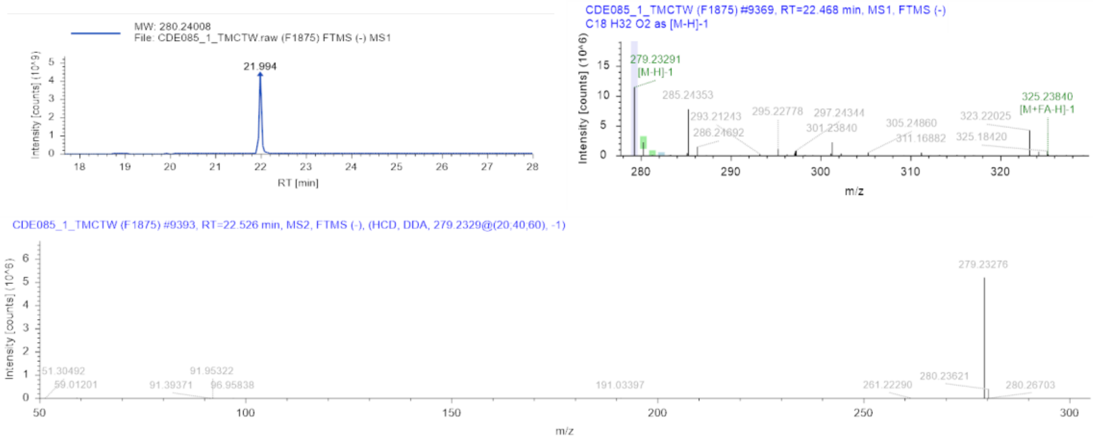

Figure S9. The mass spectrum of L-Valine.

Structure	Name	RT [min]	Formula	Calc. MW	mzCloud Best Match
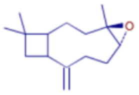	(-)-Caryophyllene oxide	15.70	C15 H24 O	220.18244	95.7998469138495

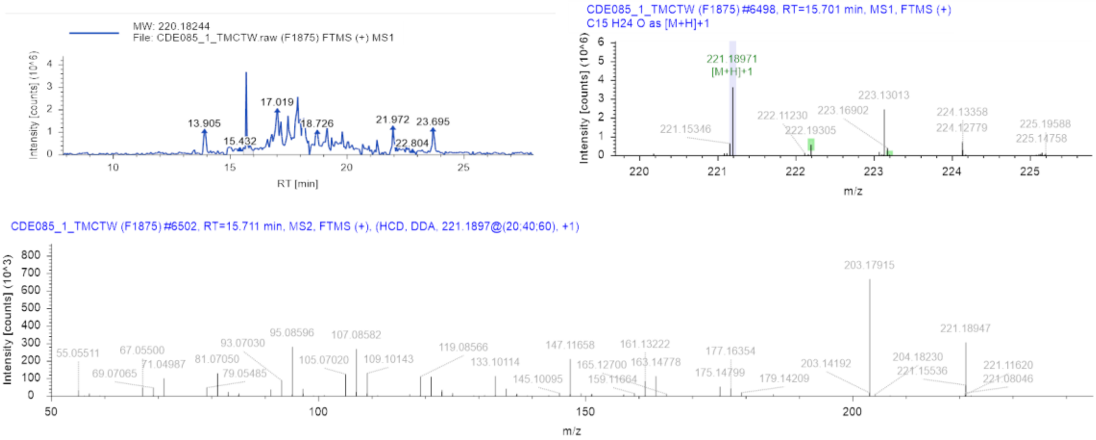

Figure S10. The mass spectrum of (-)-Caryophyllene oxide.

Structure	Name	RT [min]	Formula	Calc. MW	mzCloud Best Match
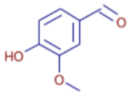	Vanillin	10.51	C8 H8 O3	152.04729	93.7076083690022

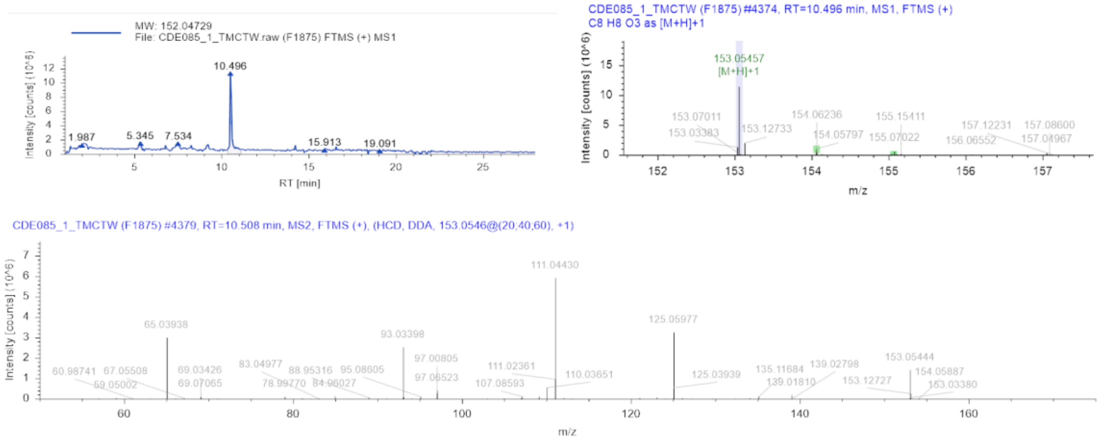

Figure S11. The mass spectrum of Vanillin.

Structure	Name	RT [min]	Formula	Calc. MW	mzCloud Best Match
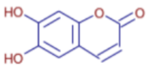	Esculetin	10.50	C9 H6 O4	196.03715	87.1923928620988

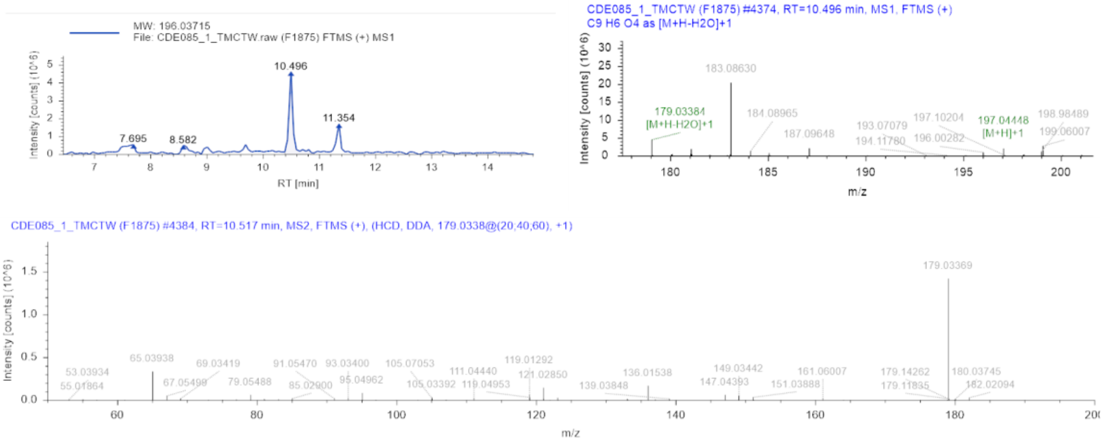

Figure S12. The mass spectrum of Esculetin.

Structure	Name	RT [min]	Formula	Calc. MW	mzCloud Best Match
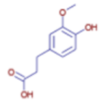	3-(4-hydroxy-3-methoxyphenyl)propanoic acid	15.65	C10 H12 O4	178.06278	**70.133283100148**

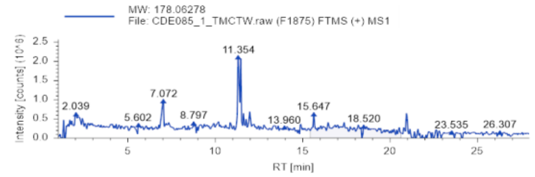

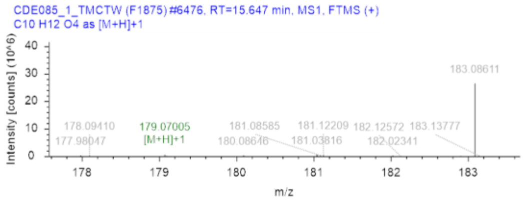

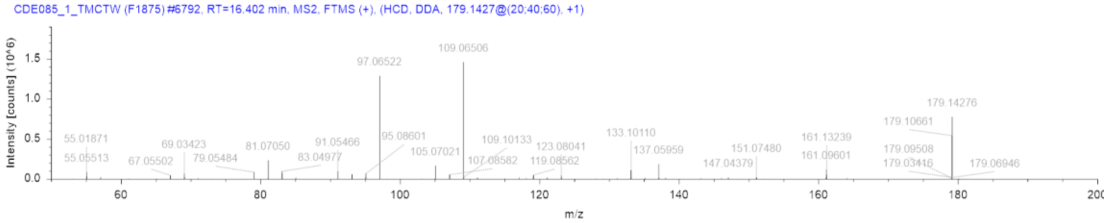

Figure S13. The mass spectrum of 3-(4-hydroxy-3-methoxyphenyl)propanoic acid.

Structure	Name	RT [min]	Formula	Calc. MW	mzCloud Best Match
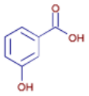	3-Hydroxybenzoic acid	8.29	C7 H6 O3	138.03053	**97.8333374025072**

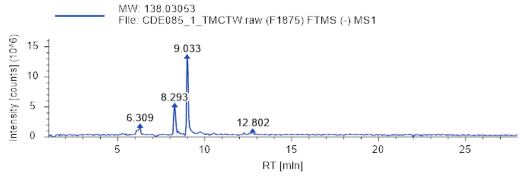

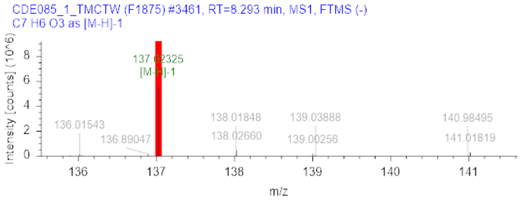

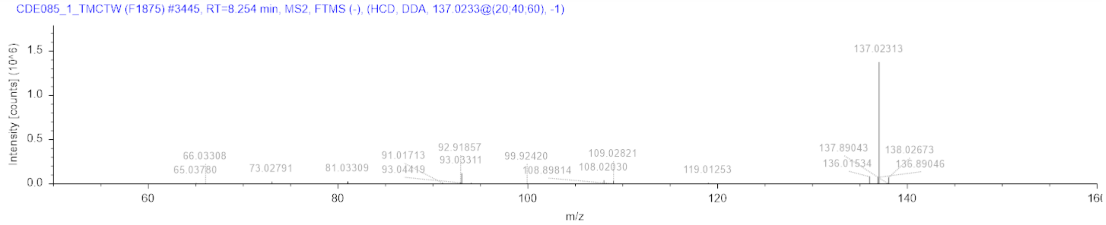

Figure S14. The mass spectrum of 3-Hydroxybenzoic acid.

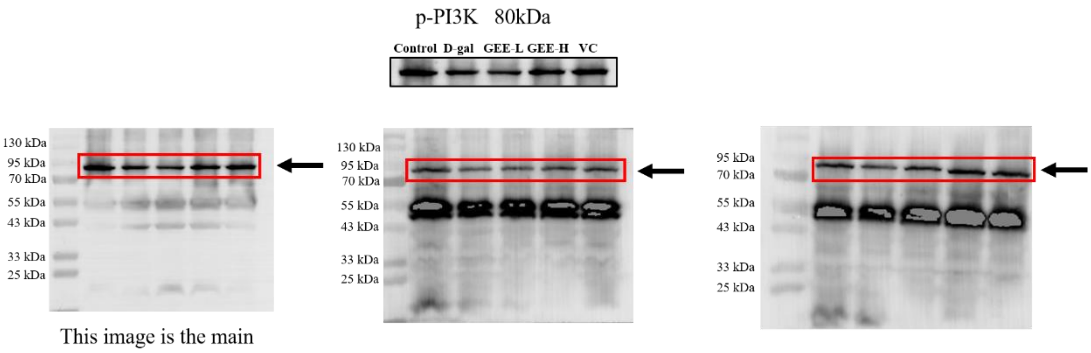

This image is the main figure used in our Western blot analysis.

Figure S15. The original Western blot analysis image of p-PI3K protein.

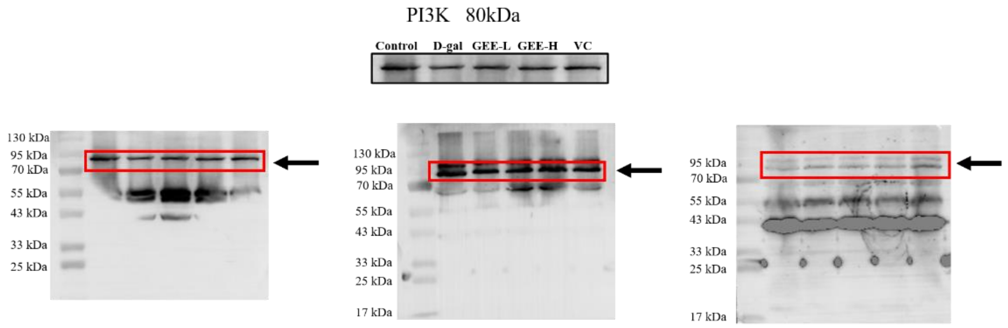

This image is the main figure used in our Western blot analysis.

Figure S16. The original Western blot analysis image of PI3K protein.

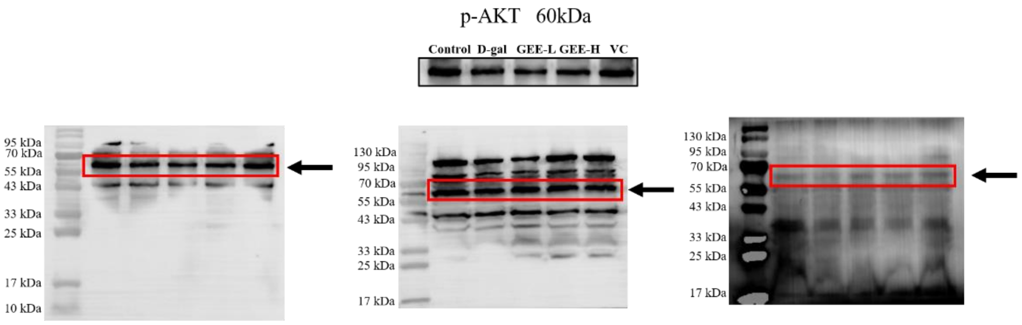

This image is the main figure used in our Western blot analysis.

Figure S17. The original Western blot analysis image of p-AKT protein.

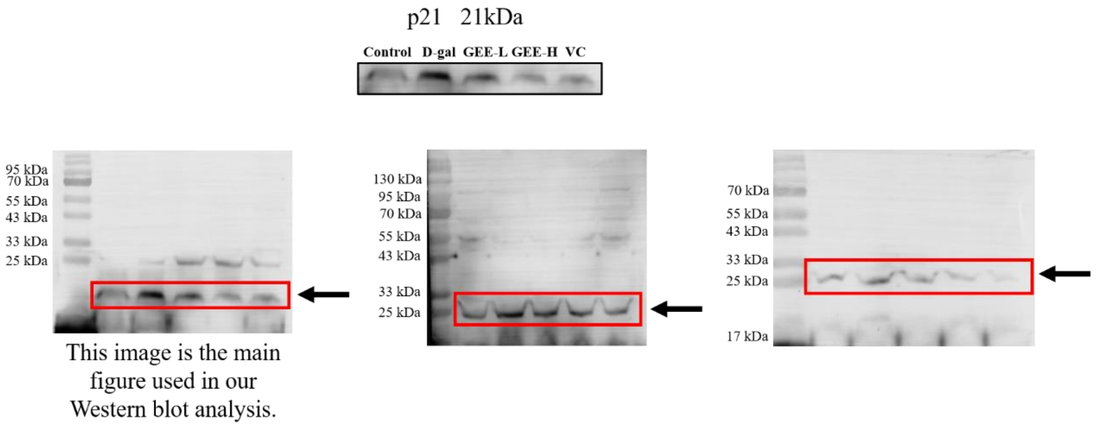

Figure S18. The original Western blot analysis image of p21 protein.

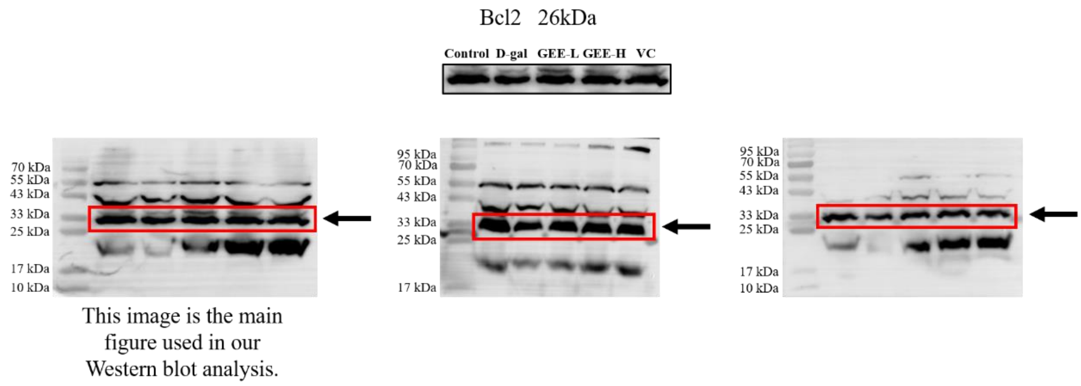

Figure S19. The original Western blot analysis image of Bcl2 protein.

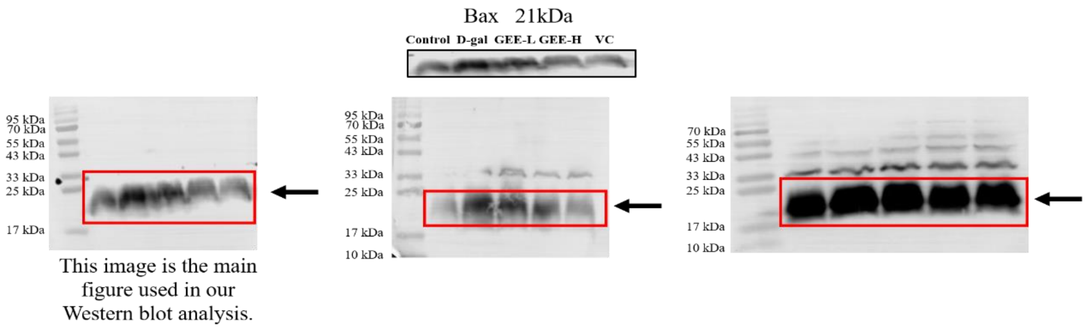

Figure S20. The original Western blot analysis image of Bcl2 protein.

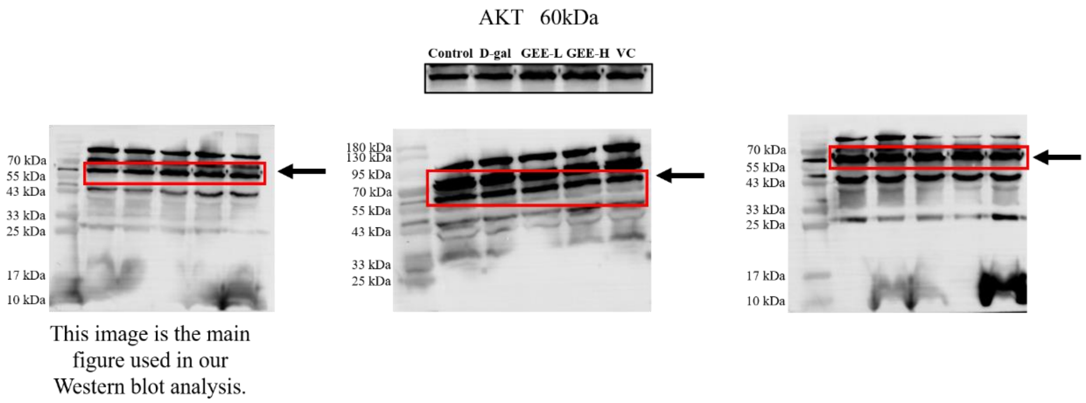

Figure S21. The original Western blot analysis image of AKT protein.

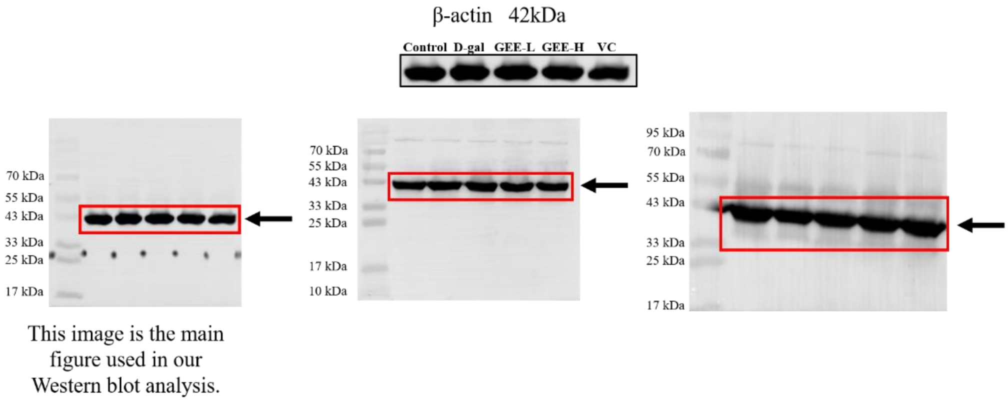

Figure S22. The original Western blot analysis image of β-actin protein.

CON group	
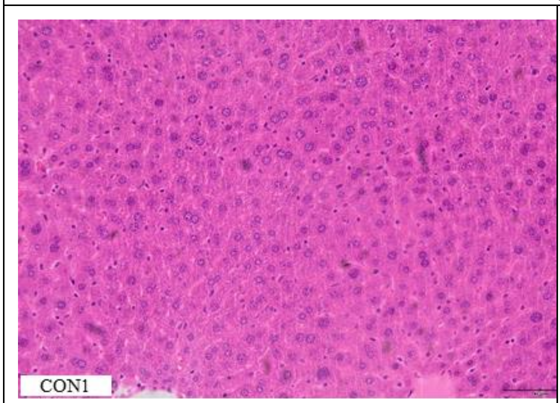	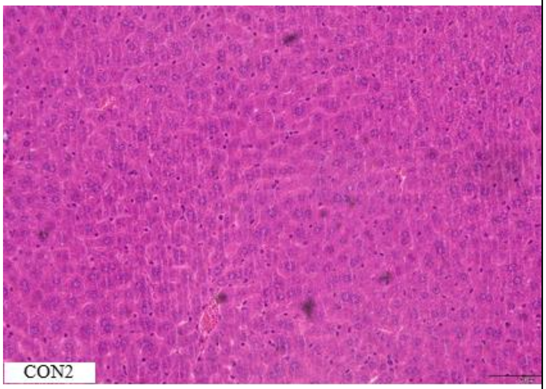
1	2

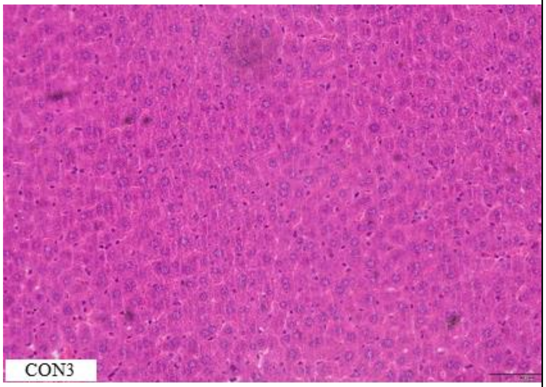	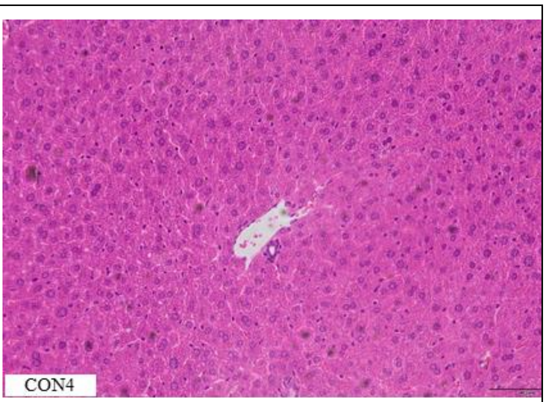
3	4
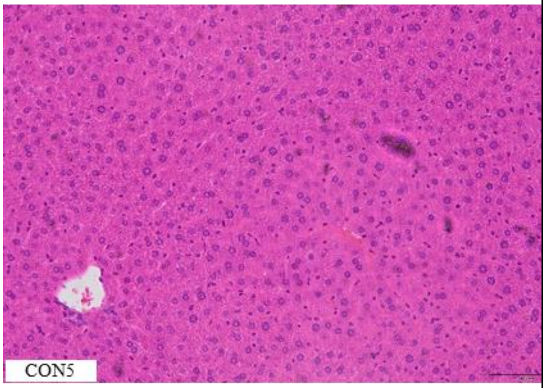	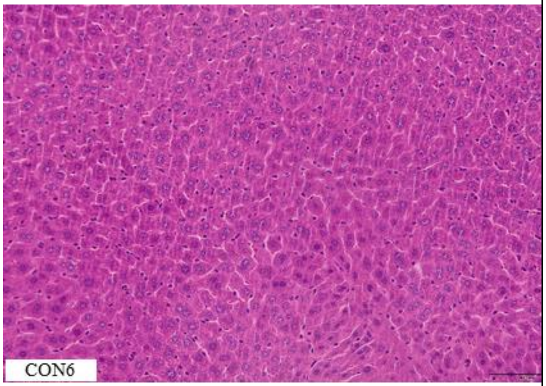
5	6
**D-gal group**	
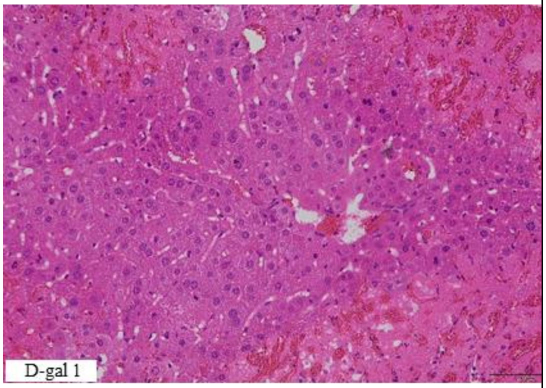	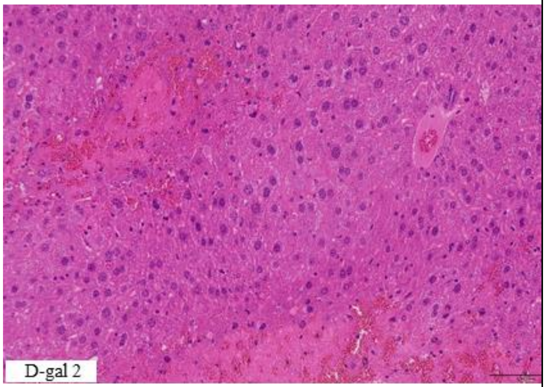
1	2
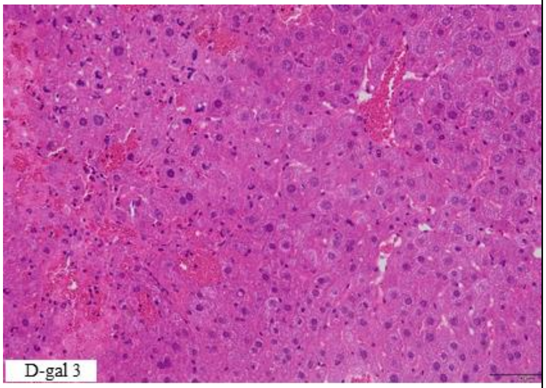	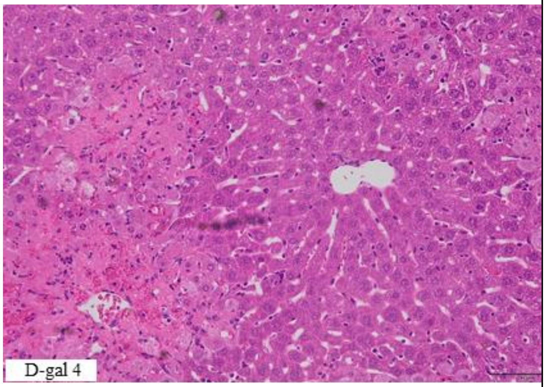

3	4
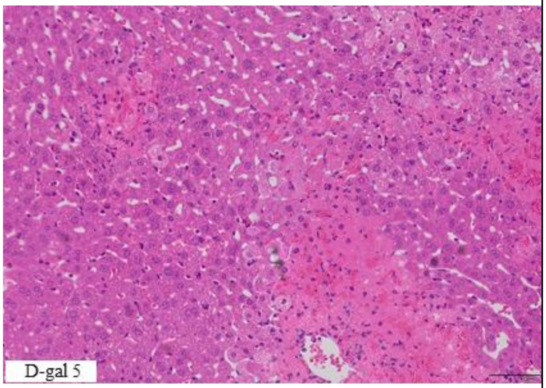	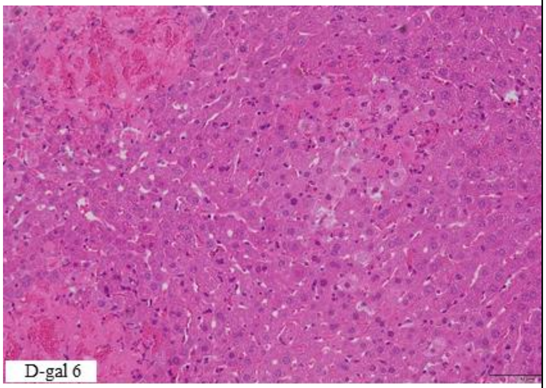
5	6
**GEE-L group**	
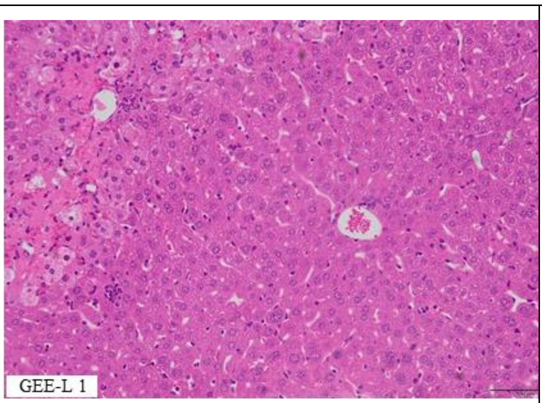	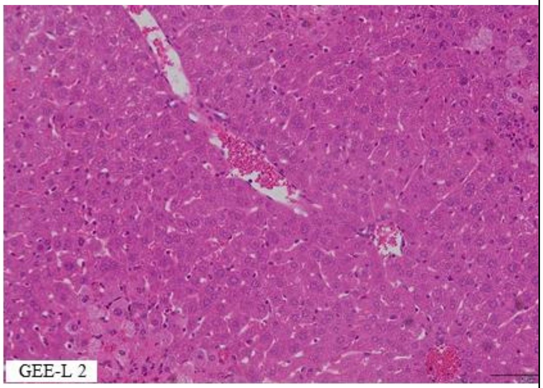
1	2
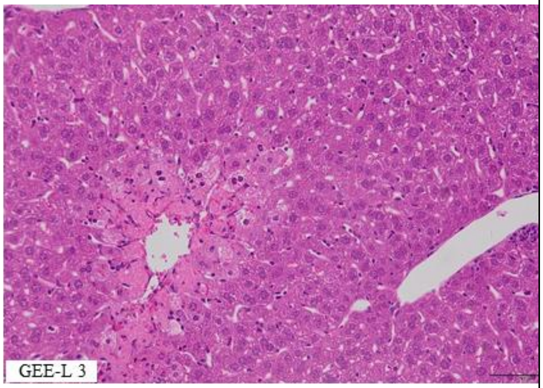	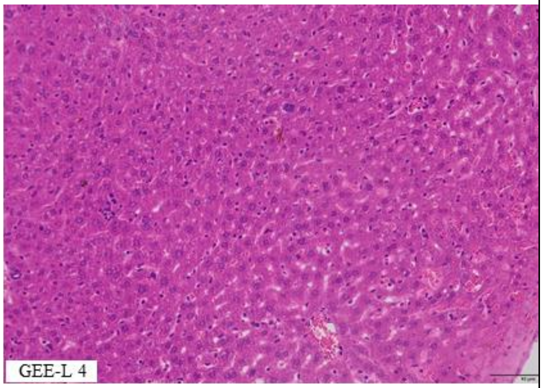
3	4

<table>
<tr><td>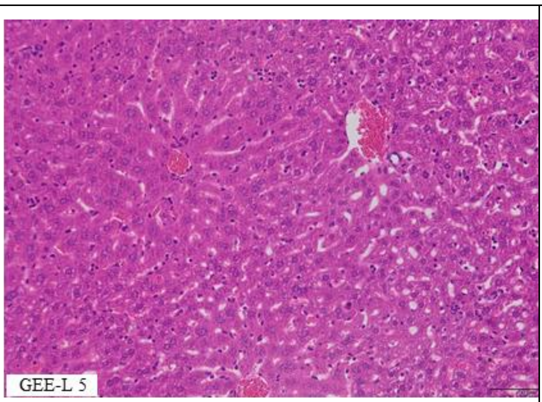
</td><td>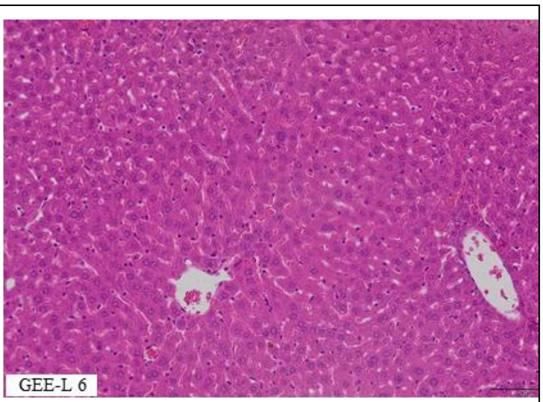
</td></tr>
<tr><td>5</td><td>6</td></tr>
<tr><td colspan="2">GEE-H group</td></tr>
<tr><td>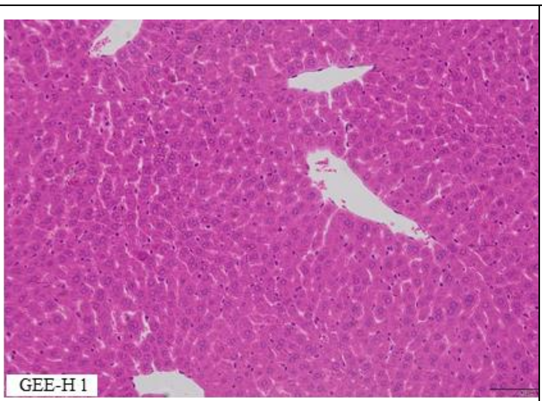
</td><td>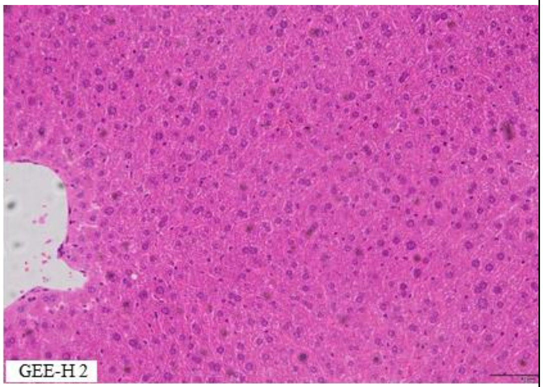
</td></tr>
<tr><td>1</td><td>2</td></tr>
<tr><td>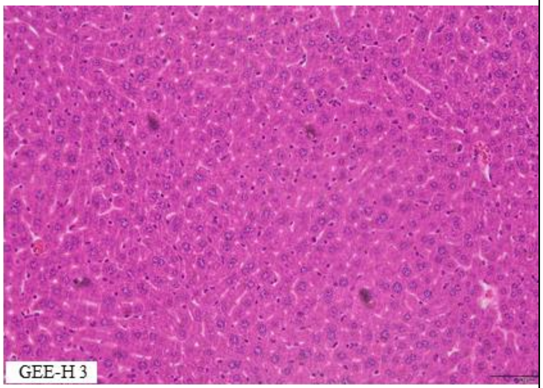
</td><td>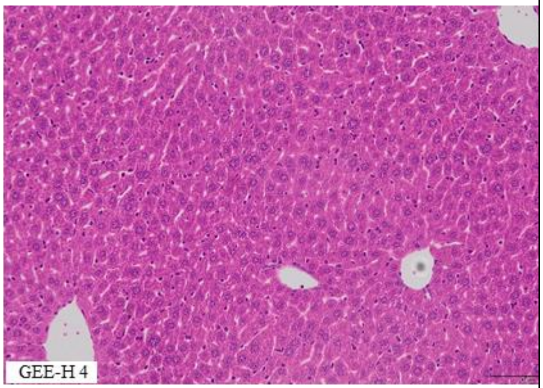
</td></tr>
<tr><td>3</td><td>4</td></tr>
<tr><td>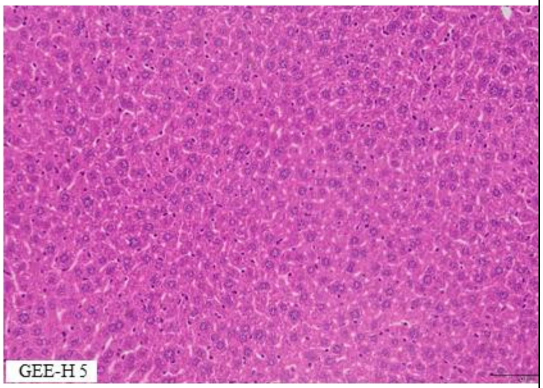
</td><td>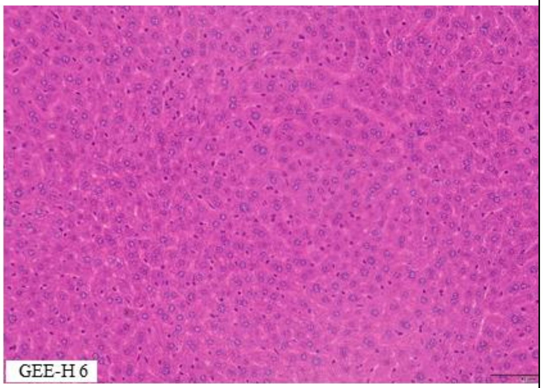
</td></tr>
</table>

<table>
<tr><td>5</td><td>6</td></tr>
<tr><td colspan="2">VC group</td></tr>
<tr><td>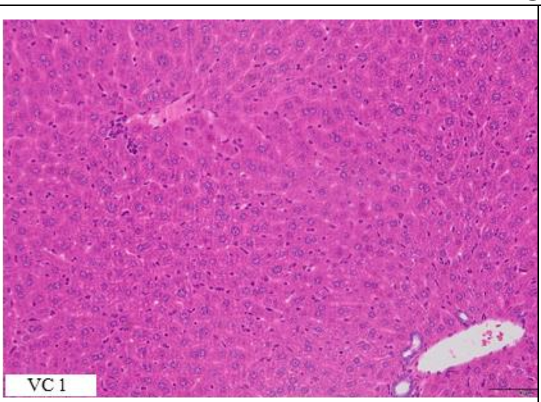</td><td>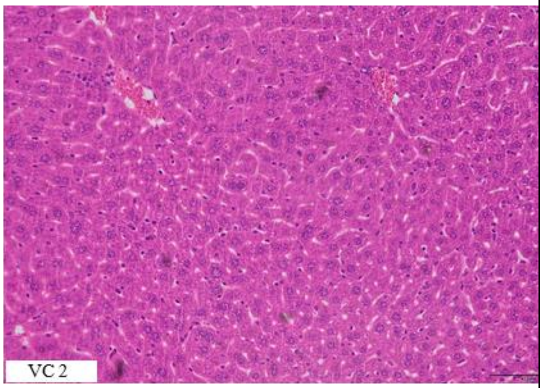</td></tr>
<tr><td>1</td><td>2</td></tr>
<tr><td>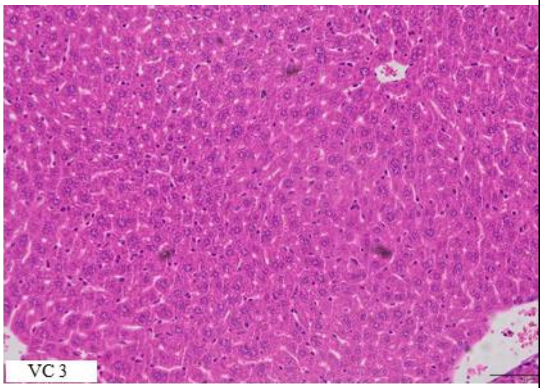</td><td>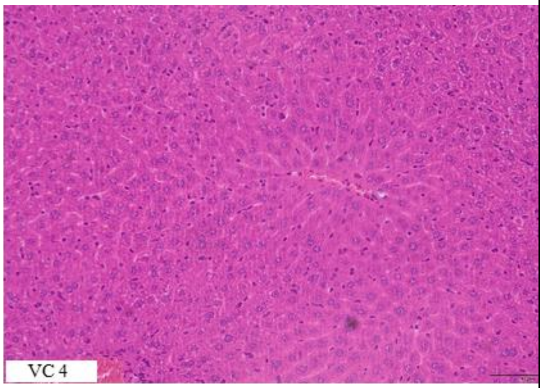</td></tr>
<tr><td>3</td><td>4</td></tr>
<tr><td>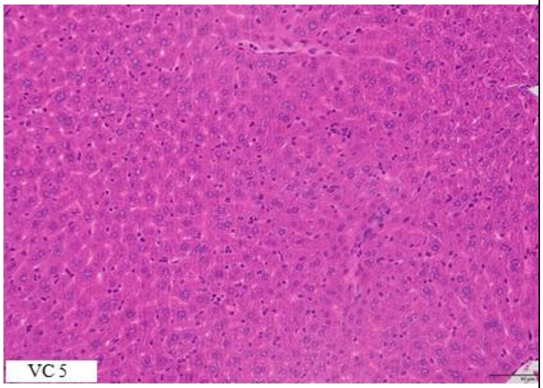</td><td>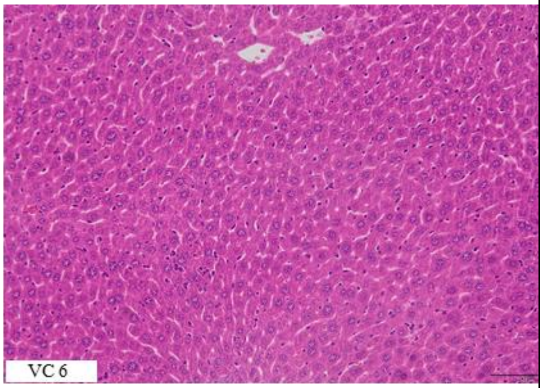</td></tr>
<tr><td>5</td><td>6</td></tr>
</table>

Figure S23. The original images of liver tissue morphology by H&E staining.

Table S1. The score results of HE staining.

Group	No.	Score
**CON**	1	0
	2	0
	3	0
	4	0
	5	0
	6	0
**D-gal**	1	3
	2	3
	3	3
	4	3
	5	3
	6	3
**GEE-L**	1	2
	2	2
	3	2
	4	1
	5	2
	6	1
**GEE-H**	1	1
	2	1
	3	1
	4	0
	5	0
	6	0
**VC**	1	1
	2	1
	3	0
	4	1
	5	1
	6	1

Score 0 indicates normal liver tissue; Score 1 indicates only a small amount of liver cell degeneration and necrosis as well as inflammatory infiltration; Score 2 indicates that the liver lobule structure is still intact, some liver cells are degenerated and necrotic, and the liver tissue shows a lot of inflammatory cell infiltration; Score 3 indicates that congestion occurs in the hepatic sinusoids and central veins, extensive liver cell degeneration and necrosis occur, the liver tissue shows a large amount of inflammatory cell infiltration, and some liver lobule structures are damaged.

Table S2. The full component identification form of GEE.

No.	Retention time [min]	Formula	Annotation MW	Calc. MW/Da	Results of identification
1	1.747	$C_6H_{13}NO_2$	131.09463	131.09459	L-Isoleucine
2	1.507	$C_9H_{11}NO_3$	165.05447	164.04717	L-Tyrosine
3	5.906	$C_6H_6O_3$	126.03169	126.03177	5-Hydroxymethyl-2-furaldehyde
4	23.826	$C_{18}H_{36}O_2$	284.27153	284.27144	Stearic acid
5	22.739	$C_{18}H_{34}O_2$	282.25588	282.25546	Oleic acid
6	20.81	$C_{18}H_{32}O_2$	280.24023	280.24006	Linoleic acid
7	1.271	$C_4H_7NO_4$	133.03751	133.03731	D-(-)-Aspartic acid
8	1.598	$C_5H_{11}NO_2$	117.07898	117.07919	L-Valine
9	15.703	$C_{15}H_{24}O$	220.18272	220.18244	(-)-Caryophyllene oxide
10	10.511	$C_8H_8O_3$	152.04734	152.04729	Vanillin
11	10.498	$C_9H_6O_4$	197.04448	196.03715	Esculetin
12	15.651	$C_{10}H_{12}O_4$	179.07005	178.06278	3-(4-hydroxy-3-methoxyphenyl)propanoic acid
13	6.297	$C_7H_6O_3$	138.03169	138.03055	3-Hydroxybenzoic acid
14	9.931	$C_9H_{12}O_3$	168.07864	150.0679	1-(4-hydroxyphenyl)propane-1,2-diol
15	1.107	$C_6H_{14}N_4O_2$	174.11168	174.11136	L-(+)-Arginine
16	14.549	$C_{10}H_{10}O_3$	178.06299	160.05214	4-Methoxycinnamic acid
17	15.406	$C_{15}H_{20}O$	184.15142	184.12481	(+)-ar-Turmerone
18	7.784	$C_9H_{10}O_4$	164.05791	164.04716	3-(3,4-dihydroxyphenyl)propanoic acid
